# Senescence-Associated Secretory Phenotypes Reveal Cell-Nonautonomous Functions of Oncogenic RAS and the p53 Tumor Suppressor

**DOI:** 10.1371/journal.pbio.0060301

**Published:** 2008-12-02

**Authors:** Jean-Philippe Coppé, Christopher K Patil, Francis Rodier, Yu Sun, Denise P Muñoz, Joshua Goldstein, Peter S Nelson, Pierre-Yves Desprez, Judith Campisi

**Affiliations:** 1 Life Sciences Division, Lawrence Berkeley National Laboratory, Berkeley, California, United States of America; 2 Buck Institute for Age Research, Novato, California, United States of America; 3 Division of Human Biology, Fred Hutchinson Cancer Research Center, Seattle, Washington, United States of America; 4 California Pacific Medical Center Research Institute, San Francisco, California, United States of America; Cancer Research UK, United Kingdom

## Abstract

Cellular senescence suppresses cancer by arresting cell proliferation, essentially permanently, in response to oncogenic stimuli, including genotoxic stress. We modified the use of antibody arrays to provide a quantitative assessment of factors secreted by senescent cells. We show that human cells induced to senesce by genotoxic stress secrete myriad factors associated with inflammation and malignancy. This senescence-associated secretory phenotype (SASP) developed slowly over several days and only after DNA damage of sufficient magnitude to induce senescence. Remarkably similar SASPs developed in normal fibroblasts, normal epithelial cells, and epithelial tumor cells after genotoxic stress in culture, and in epithelial tumor cells in vivo after treatment of prostate cancer patients with DNA-damaging chemotherapy. In cultured premalignant epithelial cells, SASPs induced an epithelial–mesenchyme transition and invasiveness, hallmarks of malignancy, by a paracrine mechanism that depended largely on the SASP factors interleukin (IL)-6 and IL-8. Strikingly, two manipulations markedly amplified, and accelerated development of, the SASPs: oncogenic RAS expression, which causes genotoxic stress and senescence in normal cells, and functional loss of the p53 tumor suppressor protein. Both loss of p53 and gain of oncogenic RAS also exacerbated the promalignant paracrine activities of the SASPs. Our findings define a central feature of genotoxic stress-induced senescence. Moreover, they suggest a cell-nonautonomous mechanism by which p53 can restrain, and oncogenic RAS can promote, the development of age-related cancer by altering the tissue microenvironment.

## Author Summary

Cells with damaged DNA are at risk of becoming cancerous tumors. Although “cellular senescence” can suppress tumor formation from damaged cells by blocking the cell division that underlies cancer growth, it has also been implicated in promoting cancer and other age-related diseases. To understand how this might happen, we measured proteins that senescent human cells secrete into their local environment and found many factors associated with inflammation and cancer development. Different types of cells secrete a common set of proteins when they senesce. This senescence-associated secretory phenotype (SASP) occurs not only in cultured cells, but also in vivo in response to DNA-damaging chemotherapy. Normal cells that acquire a highly active mutant version of the RAS protein, which is known to contribute to tumor growth, undergo cellular senescence, and develop a very intense SASP, with higher levels of proteins secreted. Likewise, the SASP is more intense when cells lose the functions of the tumor suppressor p53. Senescent cells promote the growth and aggressiveness of nearby precancerous or cancer cells, and cells with a more intense SASP do so more efficiently. Our findings support the idea that cellular senescence can be both beneficial, in preventing damaged cells from dividing, and deleterious, by having effects on neighboring cells; this balance of effects is predicted by an evolutionary theory of aging.

## Abstract 3

By controlling how damaged cells modify their surrounding tissue environment, a tumor suppressor gene can restrain, and an oncogene can promote, the development of cancer.

## Introduction

Cancer is a multistep disease in which cells acquire increasingly malignant phenotypes. These phenotypes are acquired in part by somatic mutations, which derange normal controls over cell proliferation (growth), survival, invasion, and other processes important for malignant tumorigenesis [1]. In addition, there is increasing evidence that the tissue microenvironment is an important determinant of whether and how malignancies develop [2,3]. Normal tissue environments tend to suppress malignant phenotypes, whereas abnormal tissue environments such at those caused by inflammation can promote cancer progression.

Cancer development is restrained by a variety of tumor suppressor genes. Some of these genes permanently arrest the growth of cells at risk for neoplastic transformation, a process termed cellular senescence [4–6]. Two tumor suppressor pathways, controlled by the p53 and p16INK4a/pRB proteins, regulate senescence responses. Both pathways integrate multiple aspects of cellular physiology and direct cell fate towards survival, death, proliferation, or growth arrest, depending on the context [7,8].

Several lines of evidence indicate that cellular senescence is a potent tumor-suppressive mechanism [4,9,10]. Many potentially oncogenic stimuli (e.g., dysfunctional telomeres, DNA damage, and certain oncogenes) induce senescence [6,11]. Moreover, mutations that dampen the p53 or p16INK4a/pRB pathways confer resistance to senescence and greatly increase cancer risk [12,13]. Most cancers harbor mutations in one or both of these pathways [14,15]. Lastly, in mice and humans, a senescence response to strong mitogenic signals, such as those delivered by certain oncogenes, prevents premalignant lesions from progressing to malignant cancers [16–19]. Interestingly, some tumor cells retain the ability to senesce in response to DNA-damaging chemotherapy or p53 reactivation; in mice, this response arrests tumor progression [20–22].

Despite support for the idea that senescence is a beneficial anticancer mechanism, indirect evidence suggests that senescent cells can also be deleterious and might contribute to age-related pathologies [10,23–25]. The apparent paradox of contributing to both tumor suppression and aging is consistent with an evolutionary theory of aging, termed antagonistic pleiotropy [26]. Organisms generally evolve in environments that are replete with extrinsic hazards, and so old individuals tend to be rare in natural populations. Therefore, there is little selective pressure for tumor suppressor mechanisms to be effective well into old age; rather, these mechanisms need to be sufficiently effective only to ensure successful reproduction. Further, tumor suppressor mechanisms could in principle even be deleterious at advanced ages, as predicted by evolutionary antagonistic pleiotropy. Consistent with this view, senescent cells increase with age in mammalian tissues [27], and have been found at sites of age-related pathologies such as osteoarthritis and atherosclerosis [28–30]. Moreover, in mice, chronically active p53 both promotes cellular senescence and accelerates aging phenotypes [31,32].

How might senescent cells be deleterious? Senescent cells acquire many changes in gene expression, mostly documented as altered mRNA abundance, including increased expression of secreted proteins [33–41]. Some of these secreted proteins act in an autocrine manner to reinforce the senescence growth arrest [37,38,40,41]. Moreover, cell culture and mouse xenograft studies suggest that proteins secreted by senescent cells can promote degenerative or hyperproliferative changes in neighboring cells [35,39,42,43]. Thus, although the cell-autonomous senescence growth arrest suppresses cancer, factors secreted by senescent cells might have deleterious cell-nonautonomous effects that alter the tissue microenvironment. To date, a comprehensive analysis of the secretory profile of senescent cells is lacking, as is knowledge regarding how this profile varies with cell type or senescence inducer, or how it relates to the tumor suppressor proteins that control senescence.

To fill these gaps in our knowledge, we modified antibody arrays to be quantitative and sensitive over a wide dynamic range and defined the senescence-associated secretory phenotype (SASP). We show that this phenotype is complex, containing elements associated with inflammation and tumorigenesis, and is induced only by genotoxic stress of sufficient magnitude to cause senescence. SASPs are expressed by senescent human fibroblasts and epithelial cells in culture. Moreover, epithelial tumor cells exposed to DNA-damaging chemotherapy senesce and express a SASP in vivo. The arrays allowed us to identify two new malignant phenotypes promoted by senescent cells (the epithelial–mesenchyme transition and invasiveness), and the SASP factors responsible for them (interleukin [IL]-6 and IL-8). Strikingly, the SASP was markedly amplified by oncogenic RAS or loss of p53 function. Our results identify a mechanism by which p53 acts as a cell-nonautonomous tumor suppressor, and RAS as a cell-nonautonomous oncogene, and provide a novel framework for understanding how age-related cancers might progress.

## Results

### Senescence-Associated Secretory Phenotypes Expressed by Human Fibroblasts

To determine whether tissue of origin, donor age, or genotype affected secretory phenotypes, we first studied five human fibroblast strains, derived from embryonic lung (WI-38, IMR-90), neonatal foreskin (BJ, HCA2), or adult breast (hBF184). We cultured the cells under standard conditions, and either atmospheric (∼20%) O_2_ or 3% O_2_, which is more physiological [44]. We made presenescent (PRE) cultures (>80% of cells capable of proliferation) quiescent by growing the cells to confluence in order to compare them to nondividing senescent (SEN) cultures (<10% proliferative) (Table S1). We induced senescence by either repeatedly passaging the cells (REP, replicative exhaustion) or by exposing them to a relatively high dose (10 Gy) of ionizing radiation (XRA [X-irradiation]) (see Materials and Methods; Table S1; and Text S1).

**Figure 1:**
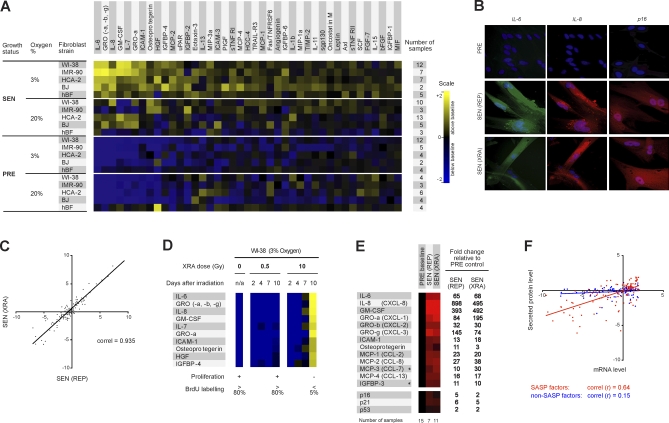
SASP of Human Fibroblasts (A) Soluble factors secreted by the indicated cells were detected by antibody arrays and analyzed as described in the text, Materials and Methods; Text S2; and Datasets S1–S4. For each cell strain, the PRE and SEN signals were averaged and used as the baseline. Senescence (SEN) was induced by either repeatedly passaging the cells (REP, replicative exhaustion) or by exposing them to a relatively high dose (10 Gy) of ionizing radiation (XRA, X-irradiation); for simplification, XRA and REP signals were averaged as a single SEN signal (see also Figure 1C). Signals higher than baseline are displayed in yellow; signals below baseline are displayed in blue. The numbers on the heat map key (right) indicates log_2_-fold changes from the baseline. (B) Validation by immunostaining. PRE WI-38 cells were made senescent by REP or XRA, maintained in 10% serum until fixation, and immunostained for the cytokines IL-6 and IL-8 and the senescence marker p16INK4a. (C) Correlation between the SASPs of WI-38 cells induced to senescence by XRA or REP, compared to PRE cells, depicted as log_2_-fold changes. (D) WI-38 cells were X-irradiated at the indicated doses. CM was collected 2, 4, 7, or 10 d later, and soluble factors were analyzed by antibody arrays. PRE cells and cells irradiated with 0.5 Gy transiently arrested growth, but resumed proliferation 24–48 h after CM collection. Cells irradiated with 10 Gy became senescent, and therefore did proliferate during and beyond the course of the experiment. PRE and SEN (10 d) signals were averaged and used as the baseline. Signals higher than baseline are displayed in yellow; signals below baseline are displayed in blue. (E) PRE and SEN cells were analyzed for the indicated mRNAs by quantitative RT-PCR, and normalized to the corresponding PRE values (baselines). The senescence inducers (REP or XRA) are given in parentheses. Signals higher than baselines are shown in red, signals below baselines are displayed in green, and the fold changes in signals are given to the right of the heat map. The results are averages for four cell strains (WI-38, IMR90, HCA-2, and BJ), and the total number of samples for each condition are given below the heat map. An asterisk (*) indicates non-SASP factors (see Figure S4). (F) Correlation between mRNA and secreted protein levels for SASP (red) and non-SASP (blue) factors (see Figure S4). PRE and SEN values were averaged to create a baseline; all values were expressed as the log_2_-fold change relative to this baseline; PRE versus baseline and SEN versus baseline are shown.

To identify proteins secreted by PRE and SEN cells, we generated conditioned media (CM) by incubating each culture in serum-free medium for 24 h. After normalizing for cell number, we analyzed CM using antibody arrays designed to detect 120 proteins selected for roles in intercellular signaling (Table S2). We modified the detection protocol (see Materials and Methods and Text S1), thereby rendering the arrays linear over two to three orders of magnitude; accurate, as determined by concordance with enzyme-linked immunosorbent assays (ELISAs) of recombinant proteins; and reliable, as determined by comparing triplicate samples analyzed separately to pooled samples (Text S2). We quantified the signals, normalizing intensities to controls on the arrays to facilitate interexperiment comparisons, and calculated secreted protein levels as log_2_-fold changes relative to averages of all samples for each cell strain (baseline). We used these values for quantitative data analyses (Datasets S1–S4) and for visual display (Figure 1A). For the visual display, values over baseline are displayed in grades of yellow, and values under baseline are displayed in grades of blue (Figure 1A).

Although the display gives only a semiquantitative assessment of how secretion levels vary (see accompanying scale in Figure 1A, with log_2_-fold changes indicated), the data (Datasets S1–S4) show that SEN cells secrete significantly higher levels of numerous proteins compared to PRE cells (Figure 1A). We term this phenomenon a senescence-associated secretory phenotype (SASP). Of 120 proteins interrogated by the arrays, 41 were significantly altered in the SEN CM and were oversecreted in comparison to PRE CM (Figure 1A and Dataset S4). However, the SASPs did not result from a general stimulation of secretion. Seventy-nine proteins showed no significant differences in secreted levels between SEN and PRE cells, although many of these proteins were easily detectable by the arrays (Dataset S4).

The SASPs were complex, and their biological effects could not be predicted a priori. SASP components included inflammatory and immune-modulatory cytokines and chemokines (e.g., IL-6, −7, and −8, MCP-2, and MIP-3a). They also included growth factors (e.g., GRO, HGF, and IGFBPs), shed cell surface molecules (e.g., ICAMs, uPAR, and TNF receptors), and survival factors (Figure 1A and Table S2). However, the SASP was not a fixed phenotype. Rather, it was a wide-ranging profile because each cell strain also displayed unique quantitative or qualitative features. In addition, within strains, PRE cultures secreted higher levels of some factors in 20% versus 3% O_2_, and SEN cultures secreted higher levels of some factors in 3% versus 20% O_2_. Thus, although human cells are less sensitive than mouse cells to hyperphysiological O_2_ [44], human cells are not unaffected by the ambient O_2_ level. Nonetheless, the secretory phenotype was highly conserved (*r* > 0.75) in human senescent cells cultured in 3% versus 20% O_2_. By contrast, the ambient O_2_ level strongly affects the secretory phenotype of mouse senescent cells (J. P. Coppe, C. K. Patil, F. Rodier, A. Krtolica, S. Parrinello, et al., unpublished data).

We verified the secretion levels of several SASP proteins by ELISAs (Figure S1 and Text S1). Further, because secretion increased greater than 10-fold for some SASP factors, we could verify up-regulation by intracellular immunostaining. For example, IL-6 and IL-8 were barely visible in PRE cells but clearly detectable in SEN cells (Figure 1B, Figure S2, and Text S1). We performed the immunostaining on cells in 10% serum, which allowed us to rule out the possibility that the SASP was a senescence-specific response to the serum-free incubation needed to collect CM. Moreover, the SASPs of SEN cells induced by REP and XRA were highly correlated (*r* > 0.9; Figure 1C), indicating that the phenotype was not specific to one senescence inducer. The secretory profiles of fibroblast strains from the same tissues (e.g., BJ and HCA2 from neonatal foreskin; and IMR90 and Wi-38 from fetal lung) were highly correlated (Datasets S3 and S4). In subsequent figures, data from these related cell strains, as well as from REP and XRA samples from cells of the same type, were pooled and averaged in order to simplify the display.

Because REP and XRA induce senescence primarily by causing genomic damage (from telomere shortening and DNA breaks, respectively), we asked whether the SASP was a primary DNA damage response. We irradiated cells using either 0.5 or 10 Gy. As expected, both doses initiated a DNA damage response, as determined by p53 stabilization and phosphorylation (see Figure S3 and Text S1). However, cells that received 0.5 Gy transiently arrested growth for only 24–48 h before resuming growth, whereas cells that received 10 Gy underwent a permanent senescence growth arrest (Figure 1D). Antibody arrays performed on CM collected between 2 and 10 d after irradiation showed that only 10 Gy induced a SASP (Figure 1D and Figure S3). Moreover, cells that senesced owing to DNA damage developed the SASP slowly, requiring 4–7 d after irradiation before expressing a robust SASP. These findings indicate the SASP is not a DNA damage response per se. However, it is induced by DNA damage of sufficient magnitude to cause senescence, after which it requires several days to develop.

We also determined that proteins comprising the SASP were, in general, up-regulated at the level of mRNA abundance (Figure 1E and 1F, red symbols and line; Figure S4, and Text S1). However, for detectable proteins that showed little or no senescence-associated change in secretion, mRNA levels were a poor predictor of secreted protein levels (Figure 1F, blue symbols and line; and Figure S4). Thus, antibody arrays provide a more accurate assessment of the senescence-associated secretory signature than mRNA profiling.

### SASPs of Human Epithelial Cells

**Figure 2:**
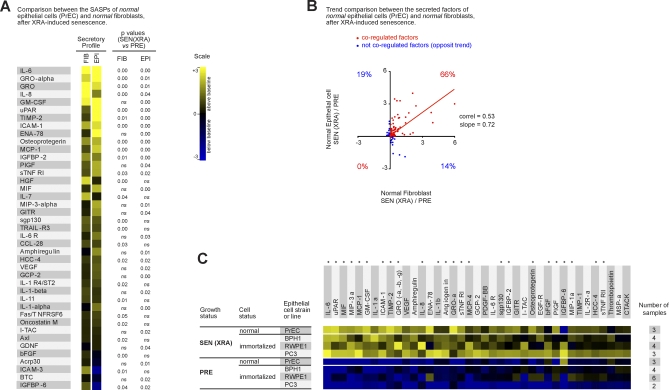
SASP of Human Epithelial Cells (A) Soluble factors secreted by the indicated normal cell type (epithelial vs. stromal) were detected by antibody arrays and analyzed as described in the text, Materials and Methods, and Datasets S5–S8. Normal prostate epithelial cells (PrECs) were induced to senesce by 10 Gy irradiation, and CM from the PRE and SEN cells were analyzed. The SASP of PrECs was compared side by side to the SASP of SEN(XRA) fibroblasts (WI-38, IMR-90, HCA-2, and BJ). The PRE values for each cell type served as the baseline. Signals higher than baseline are displayed in yellow; signals below baseline are displayed in blue. The heat map key (right) indicates log_2_-fold changes from the baseline. *p*-Values were calculated by the Student *t*-test, and are given to the right of the heat map. *ns* = not significant (*p* > 0.05) and defines non-SASP factors. (B) The log_2_-fold changes for all 120 proteins detected by the antibody arrays were plotted for SEN(XRA) PrECs and SEN(XRA) fibroblasts, relative to their PRE baseline. Seventy-nine secreted factors (66%) followed the same regulatory trend (in red). The remaining factors were not coregulated (depicted in blue). (C) Soluble factors produced by the indicated normal or transformed prostate epithelial cells were analyzed by antibody arrays and the results displayed as described for Figure 1A. For each cell strain or cell line, PRE and SEN signals were averaged and used as the baseline. Signals higher than the baseline are shown in yellow; signals below baseline are displayed in blue. An asterisk (*) indicates SASP factors that are conserved between all fibroblasts (Figure 1A) and all epithelial cells.

To determine whether the SASP is limited to fibroblasts, we studied the secretory activity of epithelial cells. Normal human prostate epithelial cells (PrECs) underwent a classic senescence growth arrest in response to X-irradiation (see Table S1). We collected CM from PRE and SEN PrECs, and analyzed the factors secreted by these cells using antibody arrays. Normal PrECs expressed a robust SASP upon senescence (Figure 2A and Datasets S5–S8). Like fibroblasts, SEN PrECs secreted many factors at significantly higher levels compared to PRE PrECs. To compare the SASPs of normal human epithelial and stromal cells senesced under similar conditions, we analyzed factors that showed a significant change (*p* < 0.05) upon senescence in PrECs or fibroblasts induced by XRA only (Figure 2A). Using the hypergeometric distribution, we determined that the SASPs of both normal cell types overlapped highly significantly (*p* = ∼10^−3^; see Materials and Methods). The trend analysis of all 120 factors on the arrays (Figure 2B) demonstrated that the secretory profiles were correlated (*r* = 0.53) and also indicated >66% overlap between normal fibroblasts and normal epithelial cells. More specifically, both SASPs included inflammatory or immune factors such as IL-6, IL-8, or MCP-1, growth modulators such as GRO and IGFBP-2, cell survival regulators such as OPG or sTNF RI, and shed surface molecules such as uPAR or ICAM-1 (Figure 2A). Not surprising, there were also differences between the SASPs of fibroblasts and PrECs. In contrast to fibroblasts, three factors (Acrp30, BTC, and IGFBP-6) were significantly down-regulated by SEN in PrECs. Moreover, IL-1α or HGF were SASP factors unique to either normal epithelial SASP or normal fibroblast SASP, respectively. This result indicates that the SASP is not limited to normal stromal cells, and that a substantial overlap between normal senescent cells of different tissue origins exists.

Some tumor cells retain the ability to senesce in response to DNA damage, including DNA-damaging chemotherapy [20–22]. We therefore asked whether prostate cancer cells also developed a SASP. We studied three prostate tumor cell lines (BPH1 [45], RWPE1 [46], and PC3 [47], which differ in their degree of malignancy as follows: PC3 > RWPE1 > BPH1). As with normal epithelial cells (PrECs), we induced senescence by XRA, and analyzed CM using antibody arrays (Datasets S5–S8). SEN epithelial cells secreted significantly higher levels of numerous proteins compared to PRE counterparts (Figure 2C). The SASPs of prostate epithelial cells showed striking overlap between normal and transformed cells (Figure 2C and Dataset S8), and there was also striking similarities between the SASP of fibroblasts and all epithelial cells, transformed and not transformed (Figure 2C, asterisks indicate common secreted proteins, and Datasets S5–S8). Twenty-four proteins were shared between the SASPs of all fibroblasts (Figure 1A) and all epithelial cells (Figure 2C); this overlap was highly significant relative to the overlap predicted from chance (*p* = ∼10^−5^; see Materials and Methods). We conclude that normal fibroblasts and both normal and transformed epithelial cells can develop SASPs that significantly overlap, displaying many common, but also some distinct, features.

### Chemotherapy-Induced SASPs Occur In Vivo

**Figure 3:**
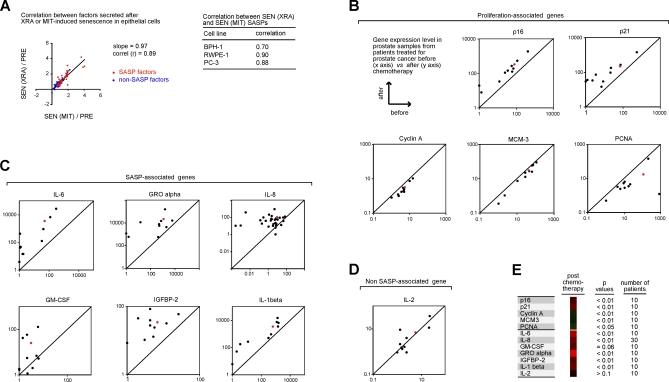
Chemotherapy-Induced SASP in Culture and In Vivo (A) Overall correlation between XRA and mitoxantrone (MIT)-induced SASPs for all three prostate epithelial cancer cell lines (BPH-1, RWPE-1, and PC-3). Correlations for the individual cell lines are given in the table to the right. The senescence inducer (XRA or MIT) is given in parentheses. (B–E) Human tumor cells were isolated from biopsies obtained from the same patient before MIT chemotherapy and from prostate tissue following prostatectomy after MIT chemotherapy. Laser captured cells were analyzed by quantitative RT-PCR for the mRNAs encoding the indicated proteins, as described in Materials and Methods and Text S1. The results are displayed on a log_10_ scale indicating the values before (horizontal or *x*-axis) compared to after (vertical or *y*-axis) chemotherapy (top left panel in [B]). Each black dot in (B, C, and D) represents the results obtained from a single patient. The average values for all patients before versus after chemotherapy are indicated by a red dot (B–D); these values are also represented as a heat map in (E). (B) Values for mRNAs encoding proteins associated with senescence (p16 and p21) and proliferation (cyclin A, MCM-3, and PCNA). (C) Values for mRNAs encoding SASP-associated proteins (IL-6, IL-8, GM-CSF, GRO-α, IGFBP-2, and IL-1β). (D) Value for an mRNA encoding a non-SASP–associated protein (IL-2). (E) Averages for the values shown in (B–D). Overall *p*-values, determined by the Student *t*-test, and number of paired samples (or patients) analyzed for each mRNA are given to the right of the heat map. Signals higher than the prechemotherapy baseline are shown in red; signals below baseline are displayed in green.

Many human tumor cells retain the ability to senesce, in culture and in vivo, in response to DNA-damaging chemotherapeutic agents [48,49]. Epithelial cell lines, as well as normal fibroblasts, underwent senescence in culture in response to mitoxantrone (MIT) (see Table S1), a topoisomerase 2β inhibitor that causes DNA breaks and is used to treat prostate cancer [50]. Antibody arrays (Figure 3A and Datasets S5–S8) and ELISAs for IL-6, IL-8, and GRO-α (Figure S1) showed that MIT induced a SASP that correlated well (*r* = 0.89) with the XRA-induced SASP (Figure 3A).

The finding that human prostatic tumor cells express a SASP in response to MIT in culture allowed us to determine whether MIT induced a SASP in vivo. We laser captured approximately 1,000 tumor epithelial cells in biopsies from human prostate cancer patients before MIT chemotherapy and in tissues removed after chemotherapy and prostatectomy [50]. By microscopic inspection, the captured cells were devoid of stromal cells and leukocytes. Since mRNA and secreted protein levels correlated well for significantly up-regulated SASP factors (Figure 1E and 1F and Figure S4), we used quantitative PCR to quantify mRNAs encoding senescence and proliferation markers and SASP factors.

After chemotherapy, most of the tumors contained significantly higher levels of p16INK4a and p21 mRNAs, which are typically up-regulated in senescent cells (Figure 3B). They also contained significantly lower levels of proliferation-associated mRNAs encoding cyclin A, MCM-3, and PCNA (Figure 3B). These results suggest that MIT induced tumor cells to senesce in vivo. Importantly, most of the tumors contained significantly higher levels of mRNAs encoding the SASP components IL-6, IL-8, GM-CSF, GRO-α, IGFBP-2, and IL-1β (Figure 3C). However, the levels of mRNA encoding IL-2, which is not a SASP component, did not significantly change on average (Figure 3D). These findings (summarized in Figure 3E) suggest that the SASP is not limited to cultured cells, but also occurs when human cells senesce in vivo.

### SASPs Induce Epithelial–Mesenchyme Transitions and Invasiveness

**Figure 4:**
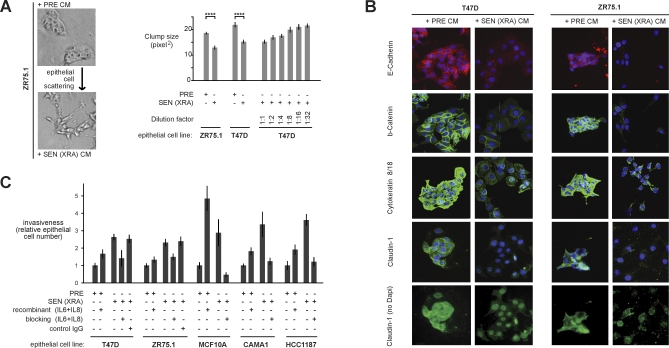
Novel SASP Biological Activities and Key Factors (A) T47D and ZR75.1 cells were incubated for 2 d with CM from PRE fibroblasts, or SEN fibroblasts induced by XRA. The cells were photographed under phase contrast, or analyzed for cluster size using an automated Cellomix imager and software. Smaller cluster or clump sizes (pixel^2^) indicate greater scattering. The senescence inducer is given in parentheses. Quadruple asterisks (****) indicate *p* < 0.001. Error bars indicate the standard deviation around the mean. (B) T47D and ZR75.1 cells were incubated with the indicated CM for 3 d and immunostained for the indicated EMT marker proteins. Induction of the mesenchymal marker vimentin by SEN (XRA) CM is shown by the western blot in Figure 7A. (C) Epithelial cell invasion was measured using Boyden chambers containing CM alone or CM plus IL-6 and IL-8 recombinant proteins or IL-6 and IL-8 blocking antibodies, as described in Materials and Methods. After 16–24 h, invasion was scored by counting the number of cells on the underside of the membrane. Invasion stimulated by PRE CM was given a value of one, and other conditions were normalized to this value. Invasion was significantly stimulated by recombinant protein and significantly inhibited by antibodies (*p* < 0.05). Error bars indicate the standard deviation around the mean.

The epithelial–mesenchymal transition (EMT) confers invasive and metastatic properties on epithelial cells, and is an important step in cancer progression that presages the conversion of carcinomas in situ to potentially fatal invasive cancers [51,52]. We found that the fibroblast SASP induced a classic EMT in two nonaggressive human breast cancer cell lines (T47D and ZR75.1). Secreted factors from SEN, but not PRE, fibroblasts caused dose-dependent epithelial cell scattering, a mesenchymal characteristic (Figure 4A). Moreover, immunostaining showed that PRE CM preserved surface-associated β-catenin and E-cadherin, and strong cytokeratin 8/18 expression (Figure 4B), and western analysis showed that PRE CM preserved low expression of vimentin (see below). These features are epithelial characteristics frequently retained by nonaggressive cells [51,52]. By contrast, CM from SEN cells markedly decreased overall and cell surface β-catenin and E-cadherin and reduced cytokeratin expression (Figure 4B), consistent with a mesenchymal transition. Further, SEN CM down-regulated the tight junction protein claudin-1, leaving the remaining protein localized primarily to the nucleus (Figure 4B), a hallmark of an EMT and a feature of metastatic but not primary tumors [53]. Finally, SEN CM increased vimentin expression (see below), another mesenchymal marker and hallmark of an EMT [52].

Consistent with a SASP-induced EMT, CM from SEN, but not PRE, cells stimulated premalignant MCF-10A and malignant T47D, ZR75.1, CAMA1, and HCC1187 cells to invade a basement membrane (Figure 4C), as well as MDA-MB-231 and MDA-MB-453 (data not shown and unpublished data). The antibody arrays guided us in identifying the highly secreted SASP components IL-6 and IL-8 as candidates for this activity [54,55]. Recombinant IL-6 and IL-8 added to PRE CM stimulated invasion to varying degrees depending on the epithelial line. Importantly, IL-6 and IL-8 blocking antibodies reduced the invasion stimulated by SEN CM (Figure 4C), indicating a substantial contribution from IL-6 and IL-8. These results support the idea that paracrine activities of the SASP can promote malignant phenotypes in nearby premalignant or malignant cells, and identify new SASP activities: the ability to induce an EMT and to invade a basement membrane.

### Oncogenic RAS Induces an Amplified SASP

Certain oncogenes, of which oncogenic RAS is the prototype [56], induce senescence in part by indirectly causing DNA damage [57,58]. RAS and related oncogenes are best known for generating mitogenic signals that promote cell-autonomous malignant phenotypes, although RAS-transformed cells are also known to secrete specific factors that contribute to tumorigenesis [59–61]. Our finding that SASPs can promote malignant phenotypes in nearby cells suggested that RAS-like oncogenes might also promote malignancy cell-nonautonomously via a complex SASP. To test this possibility, we expressed oncogenic RAS in fibroblasts and epithelial cells using lentiviruses, allowed the cells to senesce (Table S1), and analyzed CM using antibody arrays.

**Figure 5:**
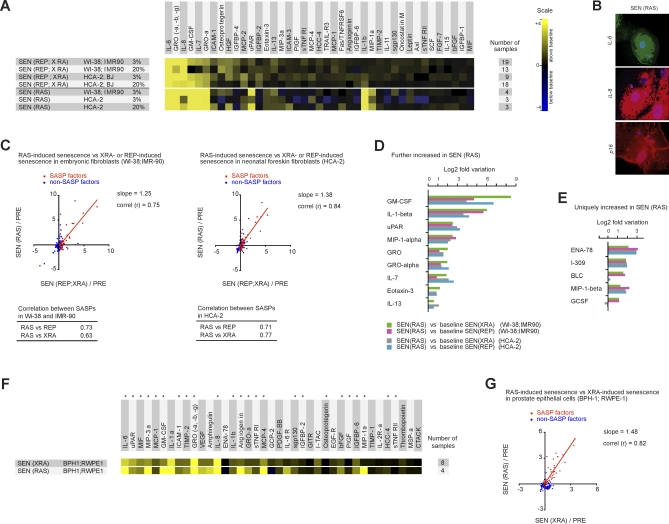
Oncogenic RAS Amplifies the SASP (A) Soluble factors produced by the indicated fibroblasts were analyzed by antibody arrays and displayed as described for Figure 1A, but in this case, PRE signals were used as the baseline. Therefore, color intensities represent log_2_-fold changes of SEN CM relative to PRE CM from cells of the same genotype under the same culture conditions. We pooled and averaged highly correlated data (see Figure 1C) from cells originating from a common tissue (WI-38, IMR-90 from embryonic lung; and HCA-2, BJ from neonatal foreskin), and from senescence induced by REP or XRA. Details of the data processing are provided in Datasets S9–S12. The senescence inducer is given in parentheses. Signals higher than baseline are shown in yellow; signals below baseline are displayed in blue. The numbers on the heat map key (right) indicates log_2_-fold changes from the baseline. (B) WI-38 cells induced to senesce by RAS were immunostained for the SASP proteins IL-6 and IL-8, and the senescence marker p16INK4a. (C) Correlations between SASPs induced by RAS versus REP or XRA in fibroblasts from embryonic lung (WI-38, IMR-90, left) or neonatal foreskin (HCA-2, right). Correlations for the individual cell strains and senescence inducers are given in the tables below the graphs. (D) Shown are the log_2_-fold values for factors that are significantly increased in CM from fibroblasts induced to senesce by RAS compared to fibroblasts induced to senesce by XRA or REP. Green indicates SEN WI-38 and IMR-90 cells induced to senesce by RAS versus XRA. Red indicates SEN WI-38 and IMR-90 induced to senesce by RAS versus REP. Gray indicates HCA2 cells induced to senesce by RAS versus XRA. Blue indicates HCA2 cells induced to senesce by RAS versus REP. (E) Shown are the log_2_-fold values for factors that are uniquely and significantly increased in CM from fibroblasts induced to senesce by RAS compared to PRE CM, but not significantly changed in CM from fibroblasts induced to senesce by XRA or REP. The color code is identical to (D). (F) Soluble factors from CM produced by the indicated epithelial cells were analyzed by antibody arrays and displayed as described for Figure 1A, using PRE CM as the baseline. Signals higher than baseline are shown in yellow; signals below baseline are in blue. Asterisks (*) indicate factors conserved between fibroblasts and epithelial cells. (G) Correlations between SASPs induced by RAS versus XRA in prostate epithelial cells.

RAS induced a SASP that had both common and unique features relative to SASPs induced by REP or XRA (Figure 5A–5E and Datasets S9–S12). The RAS-induced SASP subsumed a subset of proteins that showed increased secretion upon REP- or XRA-induced senescence (Figure 5A). To simplify the visual comparison, we averaged the highly correlated data (Figure 5A and 5C) from cells originating from the same tissue (WI-38 + IMR90 from embryonic lung, and BJ + HCA-2 from neonatal foreskin), and from cells induced to senesce by REP or XRA (see Datasets S9–S12 for details of the averaging, and the raw and processed data). Overall, there was good correlation between the SASPs of fibroblasts induced to senesce by RAS, XRA, or REP (Figure 5C). Correlations between SEN (XRA or REP) (averaged) and SEN(RAS) were 0.75 for WI-38 and IMR-90 fibroblasts (averaged) and 0.84 for HCA-2 fibroblasts.

A striking feature of the RAS-induced SASP was that all fibroblasts induced to senesce by RAS secreted multiple proteins at levels significantly and dramatically higher than other SEN cells. Because the visual display (Figure 5A) is only semiquantitative, the quantitative nature of the amplified SASP is best illustrated by the bar graph (Figure 5D), which plots the log_2_-fold increases in factors secreted by SEN(RAS) cells compared to their SEN (REP or XRA) counterparts (nine proteins were significantly more secreted in SEN(RAS) versus SEN(REP;XRA) across all fibroblasts). In addition, the RAS-induced SASPs had unique features because this SASP included five proteins that were not secreted at significantly elevated levels by other SEN (REP or XRA) cells (Figure 5E). We refer to the overall secretory response of cells induced to senesce by RAS, including the quantitative increase in secretion of specific proteins and the secretion of proteins not present in REP or XRA SASPs, as the amplified SASP. We confirmed the robust SASP induced by RAS by immunostaining (Figure 5B and Figure S2) and ELISAs (Figure S1). Further, we confirmed that oncogenic RAS induced an amplified SASP in prostate epithelial cells (Figure 5F and 5G).

Taken together, these results suggest that oncogenic RAS, despite inducing a tumor-suppressive senescence arrest, might promote tumorigenesis in a cell-nonautonomous manner by inducing an amplified SASP.

### Cell-Nonautonomous Tumor-Suppressive Function of p53

**Figure 6:**
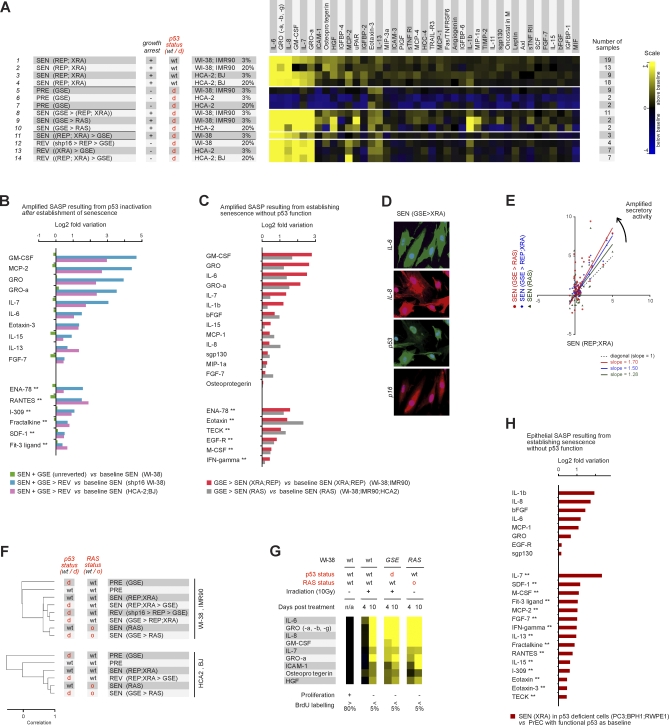
p53 Restrains the SASP (A) CM containing factors secreted by the indicted cells were analyzed by antibody arrays and displayed, using PRE CM as the baseline. We pooled data from cells of the same genotype (p53 wild type or p53 deficient) under the same culture conditions. SEN indicates pooled data from cells originating from the same tissue (WI-38, IMR-90 from embryonic lung; and HCA-2, BJ from neonatal foreskin) and induced to senesce by REP or XRA. Pooling and averaging of highly correlated samples was performed as described for Figure 5, and details of the data processing are provided in Datasets S13–S16. The top four rows are the same top four rows in Figure 5A and are included to serve as a visual reference. The senescence inducer is given in parentheses. p53 status is indicated as either wild type (wt) or deficient (d) owing to either GSE22 expression or expression of an shRNA against p53. Manipulations are indicated in sequence, separated by a greater than symbol (>). The heat map key (right) indicates the log_2_-fold changes. Signals higher than the baseline are shown in yellow; signals below baseline are displayed in blue. Comparison between rows is accurately illustrated in (B) and (C) in which each genetically manipulated cell type is compared to its appropriate control baseline. (B) Log_2_-fold values for SASP factors that are significantly increased, or significantly and uniquely (as indicated by double asterisks [**]) elevated, in CM from SEN cells made p53 deficient by GSE22, using untreated wild-type SEN values as the baseline. Green indicates WI-38 cells made senescent by XRA, after which p53 was subsequently inactivated by expressing GSE22 using a lentivirus; these cells do not resume proliferation (“unreverted”) upon p53 inactivation (see Figure S6). Blue indicates WI-38 in which p16 was inactivated by an shRNA, induced to senesce by XRA, then infected with the GSE22-expressing lentivirus; these cells do revert (REV) after p53 inactivation. Pink indicates HCA2 cells made SEN by XRA, then infected with GSE22 lentivirus; these cells also revert after p53 inactivation. (C) Log_2_-fold values for SASP factors that are significantly increased, or significantly and uniquely (as indicated by double asterisks [**]) elevated, in CM from cells made p53-deficient (by GSE22 expression), then induced to senesce by REP, XRA, or RAS. Red indicates WI-38 and IMR90 (averaged) cells expressing GSE22, then induced to senesce by XRA or REP, using cells made SEN by XRA or REP as a baseline. Gray indicates WI-38, IMR-90, and HCA2 (averaged) expressing GSE22, then made senescent by RAS, using SEN by RAS as a baseline. (D) WI-38 cells expressing GSE22 were induced to senesce by XRA and then immunostained for the SASP proteins IL-6 and IL-8, the senescence marker p16INK4a, and p53, which accumulates in the presence of GSE22. (E) Comparative graphical representation of the secretory profiles of cells made senescent by XRA or REP (dotted line), RAS (black line), p53 inactivation (GSE22) followed by XRA or REP (blue line), or p53 inactivation (GSE22) followed by RAS (red line). The increased slopes (as indicated by the arrow)indicate amplified SASPs. (F) Hierarchical cluster analysis of all the cells analyzed in (A), plus the SASP induced by RAS (see Figure 5). RAS status is indicated as either wild type (wt) or oncogenic (o) owing to expression of Ha-RAS^v12^. (G) WI-38 cells with wild-type (wt) or inactive (GSE) p53 were irradiated or induced to express oncogenic RAS (RAS), and CM was collected 4 or 10 d later. Soluble factors were analyzed by antibody arrays and displayed as described in Figure 1D, using PRE CM as the baseline (black column on the left; see also Figure S5C for details). Signals higher than baseline are shown in yellow; signals below baseline are in blue. n/a, not applicable. (H) Log_2_-fold values for prostate epithelial cell SASP factors that are significantly or uniquely (as indicated by double asterisks [**]) elevated in CM from p53-deficient cancer cells (PC3, BPH1, and RWPE1) that were induced to senesce by XRA, compared to primary p53 wild-type cells (PrECs) that were induced to senesce by XRA.

The p53 pathway is important for establishing and maintaining the senescence growth arrest caused by genotoxic stress, although cells that lack p53 can undergo senescence providing they express p16 [62]. We therefore asked whether p53 established or maintained the SASP. To inactivate p53, we expressed genetic suppressor element 22 (GSE22, also designated GSE), a peptide that prevents p53 tetramerization and causes inactive monomeric p53 to accumulate (detectable by immunostaining, Figure S2) [63]. We obtained similar results using a short hairpin RNA (shRNA) that reduces p53 expression by RNA interference. We induced WI-38 fibroblasts to senesce by REP or XRA and then inactivated p53. Because WI-38 and IMR90 cells senesce with high levels of p16INK4a, they do not resume proliferation when p53 is inactivated [62]. To simplify the visual comparison of antibody array readouts, we averaged data from highly correlated samples, as described for Figure 5 (see Datasets S13–S16 for details of the averaging, and the raw and processed data). The SASPs of SEN WI-38 in which p53 was either wild type or inactivated after senescence were similar by visual display (Figure 6A, compare row 1 with row 11), and by the graphical plot of the log_2_-fold changes that occurred in specific factors (Figure 6B, green bars showing variations obtained using the appropriate p53 wild-type baseline (e.g., SEN(REP>GSE) versus SEN(REP) in WI-38) and Datasets S13–S16). This finding indicates that p53 is not required to maintain an established SASP.

**Figure 7:**
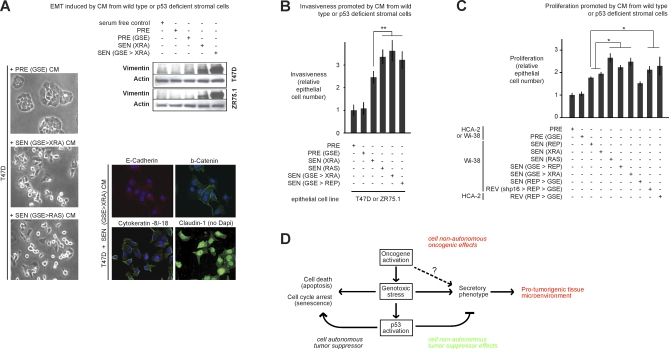
Biological Activities of the Amplified SASP (A) T47D and ZR75.1 cells were incubated with the indicated CM for 3 d and then analyzed for cell scattering, immunostained for the indicated proteins, and analyzed for vimentin and actin levels by western blotting. Controls for the immunofluorescence from the same individual experiment are shown in Figure 4B. The senescence inducer is given in parentheses. p53 status was either wild type or deficient (GSE). Manipulations are indicated in sequence, separated by >. (B) Epithelial cells were incubated with CM from the indicated WI-38 cells and assayed for invasion as described in Materials and Methods and Figure 3C. Double asterisks (**) indicate *p* < 0.02. Error bars indicate the standard deviation around the mean. (C) Epithelial cells were incubated with CM from the indicated fibroblasts and cell number was determined by cell counting, total protein, or green fluorescent protein (GFP) fluorescence using epithelial cells expressing GFP, as described in [80]. A single asterisks (*) indicates *p* < 0.05. Error bars indicate the standard deviation around the mean. (D) Model for the cell-nonautonomous activities of oncogenic RAS and the p53 tumor suppressor protein. Oncogenic and genotoxic stress of sufficient magnitude to cause senescence induce a SASP, whereby cells secrete inflammatory cytokines, chemokines, and growth factors that can alter the tissue microenvironment and stimulate malignant phenotypes in nearby cells. Thus, in addition to their well-known cell-autonomous effects, oncogenes can promote cancer cell-nonautonomously by inducing a SASP. Oncogenic and genotoxic stress also activate p53, which suppresses cancer by cell-autonomous mechanisms (promoting repair, or inducing apoptosis or senescence). In addition, p53 suppresses cancer by the cell-nonautonomous effects of suppressing the intensity of the SASP and its deleterious effects.

To determine whether p53 is needed to initiate a SASP, we inactivated p53 in PRE WI-38 cells and then induced senescence by XRA, REP, or RAS (Figure 6A, rows 8–10). p53 inactivation did not induce a SASP in PRE cells (Figures 6A, rows 5–7, Figures S1 and S2, and Datasets S13–S16). Upon senescence by either REP, XRA, or RAS, however, p53-deficient cells not only developed a SASP, but the magnitude of the SASP was markedly enhanced (Figure 6A, rows 8–10 versus 1–4), similar to the amplified SASP induced by RAS. The quantitative effect of p53 deficiency on SASPs is best illustrated by the bar graph, which plots the log_2_-fold increases of significantly altered factors secreted by cells made p53 deficient and then induced to senesce, compared to cells with wild-type p53 and induced to senesce (Figure 6C, red and grey bars). We confirmed the robust SASP by immunostaining (Figure 6D and Figure S2) and ELISA (Figure S1). Together, these findings indicate that p53 is not required to initiate the SASP, and further that it restrains development of an amplified SASP.

Importantly, the combined loss of p53 and gain of oncogenic RAS resulted in the most amplified SASP (Figure 6A, rows 9–10 versus rows 1–4; Figure 6E and 6F, Figure S5A, and Text S1). In addition, when p53 was inactivated prior to XRA, the SASP developed much earlier—between 2 and 4 d after irradiation (Figure 6G and Figures S5B and S5C), compared to 4–7 d in cells with wild-type p53 (Figure 1D). Interestingly, cells induced to senesce by RAS also developed the amplified SASP earlier—within 2–4 d after irradiation (Figures 6G, Figures S5B, S5C, and S6, and Text S1). Thus, loss of the p53 tumor suppressor, or gain of oncogenic RAS, not only amplified the SASP, but also accelerated its development.

We also inactivated p53 in fibroblasts that senesce with low p16INK4a levels: HCA2, BJ, and WI-38 expressing a shRNA (shp16) that reduces p16INK4a expression by RNA interference. In these cells, whether the cells were induced to senesce by REP or XRA, the SASP was also markedly amplified (Figure 6A, rows 1–4 versus rows 11–14). The quantitative outcome of p53 loss on established SASPs is best illustrated by the bar graph, which lists the significantly altered factors secreted by cells made to senesce and then induced to lose p53 function, compared to cells induced to senesce and keeping a functional p53 (Figure 6B, blue and pink bars). As reported [62], p53 inactivation reversed the growth arrest of these cells, and the reverted cells resumed growth (Figure S6). We refer to these cells as REV, and the reversal of the growth arrest verified the efficacy of the p53 inactivation. These findings indicate that, once established, the SASP cannot be suppressed despite reversion of the senescence growth arrest. Together, the results indicate that p53 activation by genotoxic stress not only restrains cell proliferation, but also restrains the SASP.

The SASPs of p53-deficient cells were qualitatively similar to those of SEN cells with wild-type p53, resulting in tightly clustered profiles (Figure 6F). The main influence of p53 status was quantitative. As was the case for RAS-induced senescence, a subset of SASP proteins was secreted at 5- to 30-fold higher levels after p53 inactivation (Figure 6B, 6C, and 6E, and Datasets S13–S16). However, there were also unique features of the p53-deficient SASP (Figure 6B and 6C, bottom). Interestingly, many factors that were further or uniquely up-regulated by cells made senescent by RAS were amplified in a similar fashion in p53-deficient cells (Figures 6B, 6C, 6E, 5D, and 5E).

p53 also restrained the SASP in prostate epithelial cells. Factors identified as part of the epithelial SASP (Figure 2A and 2C) were amplified in the PC3, BPH1, and RWPE1 cancer cells, which are p53 deficient, compared to normal PrECs, which have wild-type p53 (Figure 6H, top cluster). In addition, p53-deficient epithelial cells that underwent senescence oversecreted most factors that were restrained by p53 in normal fibroblasts (compare Figure 6H, bottom cluster versus listed factors in Figure 6B and 6C). Taken together, these findings indicate that fibroblasts and epithelial cells that are induced to senesce by genotoxic stress develop a SASP that is restrained by p53 activity.

### Biological Activities of Amplified SASPs

To determine the possible biological consequences of the amplified SASPs, we compared the ability of CM from fibroblasts with unamplified or amplified SASPs to induce an EMT and invasiveness in relatively nonaggressive human cancer cells. CM from cells with an amplified SASP was significantly more potent than CM from SEN cells with wild-type p53 at inducing an EMT, as determined by cell scattering (compare Figure 7A with Figure 4A), immunostaining (compare Figure 7A with Figure 4B), and robust expression of vimentin (Figure 7A), an important quantitative marker of the EMT [52]. These comparisons were made on cells from the same single experiment. Further, the amplified SASP was significantly more potent at stimulating cancer cell invasiveness (Figure 7B). Senescent cells have been shown to stimulate the growth of premalignant or malignant epithelial cells [42,43]. We found the amplified SASP stimulated this epithelial cell growth to a significantly greater extent than the unamplified SASP (Figure 7C). These findings support the idea that p53 restrains the cell-nonautonomous promalignant activities of the SASP.

## Discussion

We identified a hallmark of cellular senescence—the senescence-associated secretory phenotype or SASP—that confers cell-nonautonomous paracrine functions on cells, and is markedly exacerbated by gain of oncogenic RAS or loss of p53 function.

To study the SASP, we modified a commercially available antibody array protocol, substituting radioactivity for chemiluminescence as a final detection method. This modification greatly improved the dynamic range of the arrays, and rendered them highly quantitative, reliable, and accurate. Using this modified array protocol, we were able to study both qualitative and quantitative aspects of the SASP, and compare similarities and differences among individual donors, cell types, and tissues of origin. Importantly, we uncovered quantitative differences caused by oncogenic RAS or loss of p53 function.

The SASP was a general feature of senescent fibroblasts from different tissues, donors, and donor ages, as well as prostate epithelial cells, both normal and transformed. There were distinct quantitative and qualitative differences among the different cell strains and lines, as expected given their different genotypes and tissue origins, indicating that the SASP is not an invariant phenotype. However, the striking feature of the phenotype was the marked similarities among the SASPs from diverse donors, cell types, and tissues, suggesting the existence of a conserved core secretory program that any cell undergoing senescence would trigger. Notably, all the SASPs featured high levels of secreted inflammatory cytokines, immune modulators, and growth factors, suggesting that SASPs might have myriad biological activities in addition to those we describe here. Many SASP factors were up-regulated at the level of mRNA abundance, suggesting that the phenotype may be controlled transcriptionally.

The correspondence between mRNA levels and SASP factors allowed us to probe human biopsy samples for the expression of SASP components before and after DNA-damaging chemotherapy. Our results showed that human tumor cells very likely undergo senescence in response to DNA damaging chemotherapy in vivo, as reported for mice [49]. Moreover, human tumor cells very likely express a SASP after chemotherapy. We speculate that components of chemotherapy-induced SASPs, particularly the high levels of inflammatory cytokines, might contribute to the debilitating effects of DNA-damaging chemotherapy. These SASPs might also fuel development of secondary cancers by creating a local tissue environment that is permissive for the growth and progression of cells that acquire therapy-induced mutations, and fail to senesce or die.

Senescent human fibroblasts have been shown to stimulate the proliferation of premalignant and malignant epithelial cells in culture, and the tumorigenicity of premalignant epithelial cells in mouse xenografts [35,42,43]. However, the mechanisms responsible for these stimulatory activities are incompletely understood. We identified two new biological activities of SEN cells mediated by the SASP: the ability to induce an EMT in relatively nonaggressive carcinoma cells, and the ability to stimulate their invasion through a basement membrane. The antibody arrays allowed us to identify two SASP factors, IL-6 and IL-8, which explained much of these two biological activities. In addition to stimulating an EMT and invasiveness, IL-6 and IL-8 promote inflammation, as do other SASP components. Further, senescent cells secreted or shed cytokine receptors, which could act as decoys and allow nearby premalignant or malignant cells to avoid immune surveillance. As they persist in tissues, senescent cells likely create a proinflammatory tissue environment, which is known to be protumorigenic [3,64]. Taken together, our findings support the idea that senescent cells can create a tissue microenvironment that promotes multiple stages of tumor evolution.

Recent findings show that tumors induced to senesce in mice gradually regress [21,22], owing perhaps to infiltration by cells of the innate immune system [22]. Inflammatory cytokines and chemokines, such as IL-6, IL-8, GRO-α, MCP-1, or GM-CSF, which are core features of the SASP, might contribute to this infiltration and eventual clearance. Why, then, are senescent cells found with increasing frequency during aging and at sites of age-related pathology? Some SEN cells might be refractory to immune clearance either because they are intrinsically different or they produce higher levels of factors that promote immune evasion. Alternatively, aging or age-related pathologies may dampen immune responses or increase the rate at which senescent cells are produced. Whatever the case, there is mounting evidence that senescent cells increase with age [65–68] and that chronic inflammation is a prominent feature of aging [69]. If the senescence response is an example of antagonistic pleiotropy, the senescent microenvironment created by SASPs might contribute to degenerative diseases of aging, such as osteoarthritis or atherosclerosis [28–30], in which senescent cells are found, as well as fuel the development of late-life cancers.

The evolutionary theory of antagonistic pleiotropy provides an explanation for the apparent dilemma of how the senescence response, or any biological process, can be both beneficial and deleterious, depending on the age of the organism. It is now recognized that aging is a consequence of the declining force of natural selection with age [26,70]. This decline is due to the high mortality caused by extrinsic hazards in natural environments, resulting in the relative scarcity of older individuals. Thus, natural selection can favor a trait that contributes to early life fitness (e.g., protection from cancer), even if that trait is deleterious in older individuals (e.g., promoting cancer development). We speculate that both the growth arrest and the secretory phenotype of senescent cells can be both beneficial and deleterious.

The senescence-associated growth arrest is beneficial because it arrests the growth of cells at risk for neoplastic transformation (cell-autonomous tumor suppressor function). It can be deleterious, however, because an accumulation of nondividing senescent cells can diminish the ability of renewable tissues to repair or regenerate. Although some aged tissues contain less than one or only a few percent of senescent cells [29,66,71], others can accumulate as many as 15% senescent cells [65,72]. Likewise, the senescence-associated secretory phenotype might have both beneficial and deleterious effects. The SASP can be beneficial because some SASP components reinforce the senescent growth arrest by an autocrine cytokine network [37,38,40,41], thereby contributing to maintenance of the senescence growth arrest. In addition, many SASP components are predicted to stimulate tissue repair and regeneration, and act as “danger signals” within the vicinity of tissues or systemically at the organism level. Thus, cells undergoing senescence may initially signal tissue damage, and initiate tissue repair via the SASP. Such effect would be the beneficial cell-nonautonomous function of cellular senescence. When chronically present, however, the secretory activity of senescent cells may be deleterious, disrupting normal tissue structure and function, and eventually stimulate age-associated tissue degeneration or promote malignant phenotypes (e.g., cancer progression, as described here).

Oncogenic RAS induced a SASP that was more robust than other senescence inducers, even when p53 function was intact. Oncogenic RAS is a cell-autonomous driver of cell proliferation in many cancer cells. In normal cells, however, oncogenic RAS causes genotoxic stress and senescence [57,58], inducing a SASP and thereby conferring complex cell-nonautonomous oncogenic activities. Thus, oncogenes such as RAS, which are known to activate protumorigenic paracrine mechanisms during transformation [59–61], might also exert cell-nonautonomous protumorigenic effects through nontransformed cells during the process of inducing senescence (Figure 7D).

How does oncogenic RAS induce a SASP? One possibility is that this activity of RAS is the result of the genotoxic stress caused by RAS-stimulated hyperproliferation. Alternatively, oncogenic RAS might induce a SASP more directly by stimulating the MAP kinase or other signaling pathway. Whatever the case, many aspects of the SASP induced by RAS resembled the SASP of p53-deficient cells.

Genotoxic stress sufficient to cause senescence both activates p53 and stimulates a SASP. Our data indicate a dual role for p53 (Figure 7D). First, in responding to genotoxic stress, p53 imposes the senescence growth arrest, consistent with its role as a cell-autonomous tumor suppressor. Second, p53 restrains the SASP because loss of p53 function, in combination with senescence-causing damage, greatly amplifies the SASP. p53 might restrain the SASP in part by rapidly arresting growth after cells experience DNA damage (unpublished data), thereby preventing the accumulation of further damage that could ensue should cells attempt to replicate the damaged DNA template. Additionally, p53 optimizes DNA repair, so cells that lack p53 might accumulate more DNA damage than cells with wild-type p53, which in turn might result in a more robust (amplified) SASP. Thus, the p53 tumor suppressor may act as an early sensor of oncogenic stress, and ultimately operate as a molecular catalyst preventing tissue inflammation. The strong correlation between DNA damage and development of a SASP suggests the SASP might be activated by the mammalian DNA damage response (DDR). Indeed, our preliminary data suggest that some components of the DDR are important for establishing and maintaining the SASP (F. Rodier, J-P. Coppé, C. K. Patil, W. A. M. Hoeijmakers, D. P. Muñoz, et al., unpublished data). However, the SASP does not develop immediately after DNA damage and therefore is not a simple or classic DDR. Rather, the SASP is a slow and persistent response to severe or irreparable damage of sufficient magnitude to cause senescence.

The persistence of the SASP might have important biological consequences. For example, cells that express low p16^INK4a^ levels (e.g., SEN(REP) or SEN(XRA) HCA2) senesce in response to severe damage by activating the p53 pathway; when p53 is subsequently inactivated in these cells, they resume proliferation [62], but do not lose the SASP. Moreover, they eventually amplify the SASP as they acquire additional damage owing to proliferation in the absence of a functional checkpoint. Proliferating p53-deficient cells that senesced in response to genotoxic stress also developed a highly amplified secretory phenotype. These cells are at greater risk for escaping senescence (unpublished data) and would pose a danger to the tissue, not only by virtue of their proliferation, but also by virtue of their amplified SASP. Moreover, human cells that bypass oncogene-induced senescence [58], as well as cells in some human premalignant lesions [73,74], show signs of a persistently activated DDR. It is possible, if not likely, that these cells also express a SASP and therefore greatly increase the risk of cancer progression in vivo. By restraining the SASP, p53 acts as a cell-nonautonomous tumor suppressor, dampening the protumorigenic activities of the SASP. This activity might explain why a p53-deficient stroma promotes epithelial cancer progression [75,76]. We therefore propose that, in addition to its cell-autonomous ability to suppress cancer by inhibiting cell growth, p53 might further suppress cancer by restraining development of an inflammatory tissue milieu caused by a SASP.

Our broad, quantitative assessment of factors secreted by senescent cells revealed a highly complex secretory phenotype. We show here that this phenotype can promote cellular behaviors associated with malignancy, and suggest that cells that acquire mutations such as those that inactivate p53 and/or activate RAS functions can be particularly malignant owing to the paracrine activities of the SASP. It is very likely, though, that additional consequences of the SASP will be uncovered as the many SASP components are tested for specific activities.

## Materials and Methods

### Cells.

Cells were obtained, cultured, and made quiescent or senescent as described in Text S1 [42,66].

### Antibody arrays.

Cultures were washed and incubated in serum-free Dulbecco's modified Eagle medium (DMEM) for 24 h to generate CM, which was collected and cells counted. CM was filtered (0.2 μm pore), frozen at −80 °C, and analyzed using antibody arrays (RayBiotech or Chemicon; Human cat #AA1001CH-8; Mouse cat #AA1003M-8) essentially as per the manufacturer's instructions. Briefly, CM was thawed and concentrated 2- to 3-fold at 4 °C (3 kDa cutoff). Volumes equivalent to 2 × 10^5^ cells were diluted to 1.2 ml with DMEM and mixed with 300 μl of blocking solution. Array membranes were preincubated with 1.5 ml of blocking solution, incubated with CM mixture (overnight, 4 °C), washed 5×, then incubated with biotin-conjugated antibody cocktail (1 h 45 min, room temperature). After five washes, detection solution containing 0.265 μCi ^35^S-streptavidin (732 Ci/mmol; 0.1 mCi/ml) in blocking solution was added (1 h 45 min, room temperature), followed by five washes. Radioactivity bound to the filters was detected and quantified using a phosphorimager. Signals were analyzed as described in Text S2.

### ELISA, immunofluorescence, and invasion assays.

Assays were performed as described [77,78], using kits and antibodies described in Text S1.

### Recombinant proteins, blocking antibodies, and vectors.

Recombinant proteins and blocking antibodies were obtained as described in Text S1. Vectors to express oncogenic RAS (Ha-RAS^v12^), TIN215C, and GSE22 were described [62,77,79].

### Human study and tissues.

Patients with high-risk localized prostate cancer enrolled and treated on a phase I–II clinical neoadjuvant chemotherapy trial at the Oregon Health & Science University, Portland VA Medical Center, Kaiser Permanente Northwest Region, Legacy Health System, and University of Washington [50]. Patients provided signed informed consent. From each patient, prostate biopsies were obtained prior to chemotherapy. At the time of radical prostatectomy following chemotherapy, cancer-containing tissue samples were obtained and frozen. Frozen sections were processed as described in Text S1. Cancerous epithelium from pretreated biopsy and posttreated prostatectomy specimens were captured separately and histology of acquired cells verified by review of hematoxylin and eosin (H&E)-stained sections from each sample and review of the laser confocal microscopy (LCM) images.

### Real-time polymerase chain reaction (RT-PCR).

RNA was isolated from cultured or laser-captured cells and analyzed as described in Text S1.

### Statistical analyses.

Correlation coefficients were evaluated using Pearson correlation. Statistical significance between distributions of protein or mRNA signals was evaluated using a Student *t*-test with two tails, and an assumption of equal variance. For determination of the significance of overlap between epithelial and fibroblast SASPs, we used the hypergeometric distribution with the following parameters: population size = 120 (total proteins on the array), sample size = 41 (fibroblast SASP; see Figure 1A), successes in population = 39 (epithelial SASP; see Figure 2C), and successes in sample = 24 (overlap between the fibroblast and epithelial SASPs; see Figure 2C, asterisks). The same statistical analysis was used to compare SEN(XRA) normal epithelial cells (PrECs) versus SEN(XRA) normal fibroblasts (the following parameters were used: 120, 29, 25, and 12; see Figure 2A and Results).

## Supporting Information

Figure S1 ELISA Measurements of SASP Soluble Factors (A) Selected SASP factors (IL-6 [green], IL-8 [blue), GRO-α/KC [orange], FGF-7/KGF[(pink], and OPG [grey]) present in CM and identified by array analyses were validated and quantified by ELISAs, as described in Materials and Methods.
(B) IL-6 (green), IL-8 (blue), and GRO-α (orange) were measured in CM from SEN(XRA) and SEN(MIT) fibroblasts and epithelial cells by ELISA. Values were normalized to PRE levels.
(196 KB PDF)

Figure S2 Immunostaining for SASP Factors Selected SASP factors present in CM and detected by array analyses were visualized intracellularly by immunofluorescence. The cells were also immunostained for p16 and p53. Each panel is a different field. Cells are designated as described in the text and Table S1. Growth status indicated by a plus sign (+) means proliferating, a negative sign (−) means cell cycle arrested. p53 status indicated by a plus sign (+) means wild type, a negative sign (−) means deficient.
(582 KB PDF)

Figure S3 p53 Activation by Low- or High-Dose X-Irradiation WI-38 cells were X-irradiated with 0, 0.5, or 10 Gy X-rays. Cell lysates were prepared 2 h or 10 d later and analyzed for total p53 protein levels, p53 phosphorylated on serine 15 (p-p53ser15), or actin (loading control) by western blotting.
(117 KB PDF)

Figure S4 Comparison between Secreted Protein Levels and mRNA levels in PRE and SEN(XRA and REP) Human Fibroblasts (A) Shown are heat maps of selected SASP (secreted protein levels significantly up-regulated by SEN compared to PRE cells) and non-SASP (secreted protein levels not significantly changed by SEN compared to PRE cells) factors. Studied SASP factors are IL-6 and −8, GRO-α, -β, and -γ, GM-CSF, ICAM-1, OPG, MCP-1, −2, and −4, and leptin. Studied non-SASP factors are MCP-3, RANTES, ENA-78, PDGF-B, IGFBP-3, eotaxin, GCP-2, and AREG. The left column lists the factors, all of which were readily detected by the antibody arrays (see Dataset S4). The right column gives the correlation between mRNA and secreted protein levels. For each of the indicated cell strains (WI-38, IMR-90, HCA-2, and BJ), the colored display shows the average mRNA level (green below baseline; red above baseline) or average secreted protein level (blue below baseline; yellow above baseline), all relative to the average for all samples from each strain. Some of the SASP components show a high correlation between mRNA and secreted protein level. However some of the SASP and almost all of non-SASP factors show a poor or even negative correlation between the secreted protein and mRNA level.
(B) Overall comparison between secreted protein levels and mRNA levels in PRE and SEN (XRA and REP). All PRE and SEN measurements were averaged to create a baseline, as described in (A). All data points presented in (A) are plotted.
(C) PRE and SEN data points are plotted separately. Each plot shows all SASP and non-SASP factors.
(D) SASP and non-SASP data points are plotted separately. Each plot shows all PRE and SEN data points.
(371 KB PDF)

Figure S5 The SASP Development Is Regulated by p53 and RAS (A) Comparative analysis of SASPs extent using WI-38 and IMR90 as a model for senescence establishment and maintenance. The senescence inducer is given in parenthesis. The p53 and RAS status are listed below (a plus sign [+] means wild type; a negative sign [−] means dysfunctional for p53 and oncogenic for RAS). The secretory profile extent is the number of significantly oversecreted factors composing each SASP. Using the SEN (REP;XRA) profile as the baseline SASP profile (p53 and RAS wild type), the other SASPs can be analyzed as follow: some secreted factors are overall conserved and overlap with the SEN (REP;XRA) SASP; among these conserved factors, some are significantly further increased; finally, some other factors are unique to these other SASPs (see also Figures 5 and 6). This comparison shows that the loss of p53 tumor suppressor combined with the gain of oncogenic RAS allow the development of the most amplified SASP.
(B) WI-38 cells, wild-type (wt), or p53-deficient (expressing GSE; noted as a minus sign [−]), were irradiated at the indicated doses or induced to senescence by RAS (see also Figures 1D and 6G). CM were collected 2, 4, 7, or 10 d later, and soluble factors were detected and displayed. PRE and SEN (10 d) signals were averaged and used as the baseline. Signals higher than baseline are shown in yellow; signals below baseline are in blue (see Figure 1A and 1D, legend).
(C) Unsupervised hierarchical cluster analysis of cells presented in Figure 6G. PRE cells secretory profile was compared to the secretory profile of cells whose CM was collected at 4 d or 10 d after low-dose (0.5 Gy) or high-dose (10 Gy) irradiation, or induced to senescence due to oncogenic RAS overexpression, and lacking or not p53 function (GSE). Note that cells that senesced while lacking p53 function or due to oncogenic RAS overexpression cluster together at each time point after senescence induction. Cells irradiated with 0.5 Gy are very similar to PRE at all time points postdamage (correlation > 0.95). At 4 d post 10 Gy irradiation, cells harboring a wild-type p53 pathway are still very similar to their PRE counterpart (correlation > 0.95), whereas at 10 d post 10 Gy irradiation, cells are very dissimilar to PRE (correlation < 0 ; they have developed a SASP). The clustering analysis also shows that cells that senesced in the absence of p53 function or due to oncogenic RAS overexpression resemble more each other than cells that senesced with a wild-type p53 background, suggesting that the loss of p53 and the gain of oncogenic RAS have similar dominant effects over SASP development and establishment.
(142 KB PDF)

Figure S6 Growth Reversion of SEN Cells after p53 Inactivation SEN(REP) and SEN(XRA) WI-38 cells were monitored for cell growth for 20 d before infection with lenti-GSE (rectangle). Cell number was subsequently monitored for an additional 30 d thereafter. Because SEN WI-38 cells express p16, p53 inactivation by GSE does not revert the SEN growth arrest. Cells that do not express p16 at SEN (shp16-expressing WI-38 or unmodified HCA2 cells) were similarly monitored and infected. In contrast to SEN WI-38 cells, p16-deficient cells resumed growth (reverted) after p53-inactivation and proliferated for at least the ensuing 30 d.
(79 KB PDF)

Dataset S1 Computational Analysis of Antibody Array Data Presented in Figure 1 (Human Fibroblasts; SEN(XRA) and SEN(REP)): Part 1 (31 KB DOC)

Dataset S2 Computational Analysis of Antibody Array Data Presented in Figure 1 (Human Fibroblasts; SEN(XRA) and SEN(REP)): Part 2 (28 KB XLS)

Dataset S3 Computational Analysis of Antibody Array Data Presented in Figure 1 (Human Fibroblasts; SEN(XRA) and SEN(REP)): Part 3 (142 KB XLS)

Dataset S4 Computational Analysis of Antibody Array Data Presented in Figure 1 (Human Fibroblasts; SEN(XRA) and SEN(REP)): Part 4 (162 KB XLS)

Dataset S5 Computational Analysis of Antibody Array Data Presented in Figure 2 and Figure 3 (Human Epithelial Cells; SEN(XRA)): Part 1 (31 KB DOC)

Dataset S6 Computational Analysis of Antibody Array Data Presented in Figure 2 and Figure 3 (Human Epithelial Cells; SEN(XRA)): Part 2 (39 KB XLS)

Dataset S7 Computational Analysis of Antibody Array Data Presented in Figure 2 and Figure 3 (Human Epithelial Cells; SEN(XRA)): Part 3 (78 KB XLS)

Dataset S8 Computational Analysis of Antibody Array Data Presented in Figure 2 and Figure 3 (Human Epithelial Cells; SEN(XRA)): Part 4 (159 KB XLS)

Dataset S9 Computational Analysis of Antibody Array Data Presented in Figure 5 (Oncogene-Induced Senescence): Part 1 (31 KB DOC)

Dataset S10 Computational Analysis of Antibody Array Data Presented in Figure 5 (Oncogene-Induced Senescence): Part 2 (19 KB XLS)

Dataset S11 Computational Analysis of Antibody Array Data Presented in Figure 5 (Oncogene-Induced Senescence): Part 3 (90 KB XLS)

Dataset S12 Computational Analysis of Antibody Array Data Presented in Figure 5 (Oncogene-Induced Senescence): Part 4 (61 KB XLS)

Dataset S13 Computational Analysis of Antibody Array Data Presented in Figure 6 (p53-Deficient Senescence): Part 1 (31 KB DOC)

Dataset S14 Computational Analysis of Antibody Array Data Presented in Figure 6 (p53-Deficient Senescence): Part 2 (25 KB XLS)

Dataset S15 Computational Analysis of Antibody Array Data Presented in Figure 6 (p53-Deficient Senescence): Part 3 (133 KB XLS)

Dataset S16 Computational Analysis of Antibody Array Data Presented in Figure 6 (p53-Deficient Senescence): Part 4 (81 KB XLS)

Table S1 Presenescent and Senescent Cells Characteristics Labeling index and senescence-associated beta-galactosidase (SA-bGal) staining of human fibroblasts and human prostate epithelial cells in vitro.
(194 KB DOC)

Table S2 Complete Catalog of Entrez Gene IDs for All Proteins Corresponding to Antibodies on the Arrays (61 KB XLS)

Text S1 Supplemental Methods (42 KB DOC)

Text S2 Computational Processing, Analysis, and Validation of Antibody Arrays Digitization and quantification of antibody arrays; numerical and statistical methods; linearity; accuracy; and reliability.
(289 KB DOC)

## Abbreviations

CM: conditioned medium
DDR: DNA damage response
ELISA: enzyme-linked immunosorbent assay
EMT: epithelial–mesenchymal transition
GSE: genetic suppressor element
IL: interleukin
MIT: mitoxantrone
PRE: presenescent
PrEC: normal human prostate epithelial cell
REP: replicative exhaustion
SASP: senescence-associated secretory phenotype
SEN: senescent
shRNA: short hairpin RNA
XRA: X-irradiation
