# Supplementary material for: Senescence-Associated Secretory Phenotypes Reveal Cell-Nonautonomous Functions of Oncogenic RAS and the p53 Tumor Suppressor
Source: PLoS Biol. 2008 Dec 2;6(12):e301. doi: 10.1371/journal.pbio.0060301 (PMC2592359; doi:10.1371/journal.pbio.0060301)
Supplement: Supplementary file 2 [file pbio.0060301.sg002.pdf]

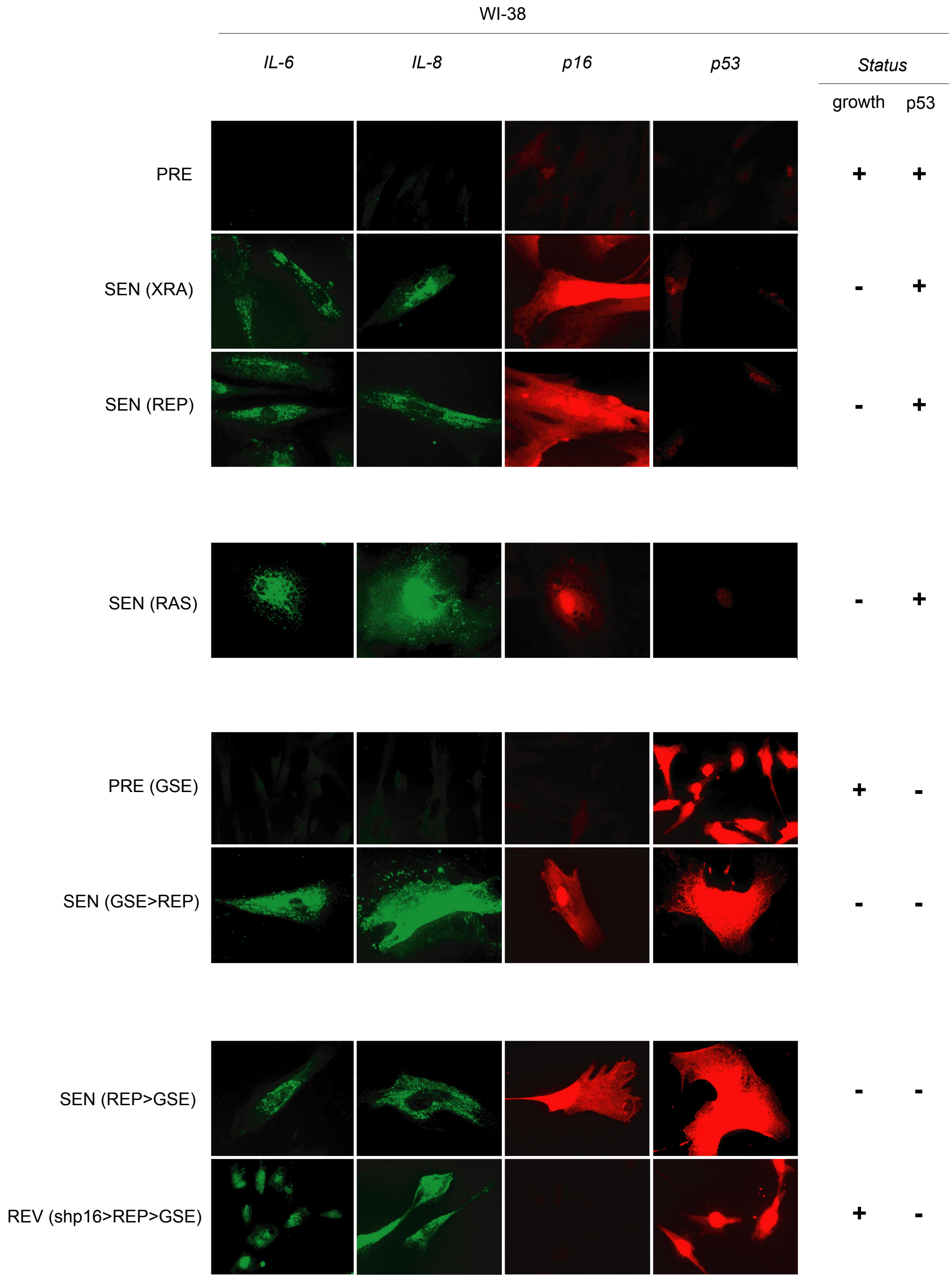
WI-38
IL-6
IL-8
p16
p53
Status
growth
p53
PRE
+
+
SEN (XRA)
-
+
SEN (REP)
-
+
SEN (RAS)
-
+
PRE (GSE)
+
-
SEN (GSE>REP)
-
-
SEN (REP>GSE)
-
-
REV (shp16>REP>GSE)
+
-
