# Supplementary material for: Senescence-Associated Secretory Phenotypes Reveal Cell-Nonautonomous Functions of Oncogenic RAS and the p53 Tumor Suppressor
Source: PLoS Biol. 2008 Dec 2;6(12):e301. doi: 10.1371/journal.pbio.0060301 (PMC2592359; doi:10.1371/journal.pbio.0060301)
Supplement: Supplementary file 3 [file pbio.0060301.sg003.pdf]

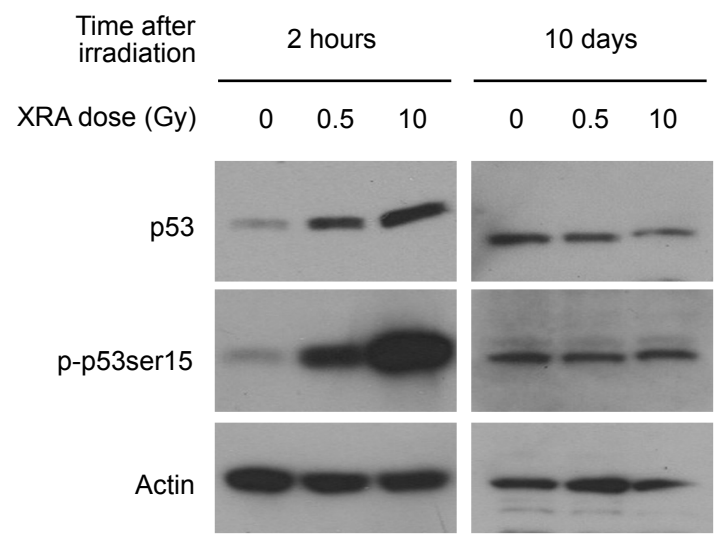
Time after irradiation
2 hours
10 days
XRA dose (Gy)
0
0.5
10
0
0.5
10
p53
p-p53ser15
Actin
