# Supplementary material for: Senescence-Associated Secretory Phenotypes Reveal Cell-Nonautonomous Functions of Oncogenic RAS and the p53 Tumor Suppressor
Source: PLoS Biol. 2008 Dec 2;6(12):e301. doi: 10.1371/journal.pbio.0060301 (PMC2592359; doi:10.1371/journal.pbio.0060301)
Supplement: Supplementary file 4 [file pbio.0060301.sg004.pdf]

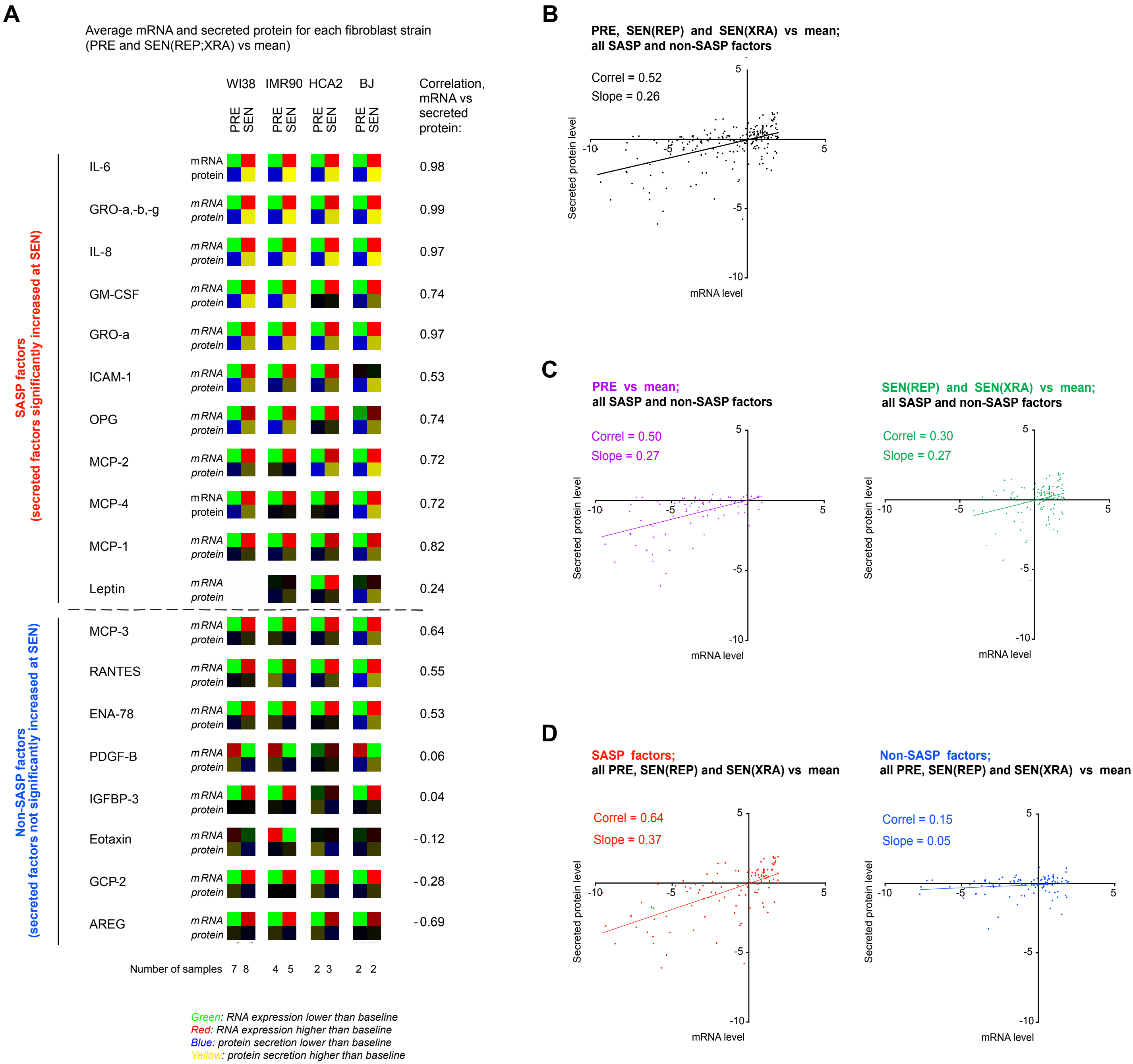
A
Average mRNA and secreted protein for each fibroblast strain
(PRE and SEN(REP;XRA) vs mean)
WI38 IMR90 HCA2 BJ
PRE SEN PRE SEN PRE SEN PRE SEN
Correlation, mRNA vs secreted protein:
SASP factors
(secreted factors significantly increased at SEN)
IL-6 mRNA protein 0.98
GRO-a,-b,-g mRNA protein 0.99
IL-8 mRNA protein 0.97
GM-CSF mRNA protein 0.74
GRO-a mRNA protein 0.97
ICAM-1 mRNA protein 0.53
OPG mRNA protein 0.74
MCP-2 mRNA protein 0.72
MCP-4 mRNA protein 0.72
MCP-1 mRNA protein 0.82
Leptin mRNA protein 0.24
Non-SASP factors
(secreted factors not significantly increased at SEN)
MCP-3 mRNA protein 0.64
RANTES mRNA protein 0.55
ENA-78 mRNA protein 0.53
PDGF-B mRNA protein 0.06
IGFBP-3 mRNA protein 0.04
Eotaxin mRNA protein -0.12
GCP-2 mRNA protein -0.28
AREG mRNA protein -0.69
Number of samples 7 8 4 5 2 3 2 2
Green: RNA expression lower than baseline
Red: RNA expression higher than baseline
Blue: protein secretion lower than baseline
Yellow: protein secretion higher than baseline
B
PRE, SEN(REP) and SEN(XRA) vs mean;
all SASP and non-SASP factors
Correl = 0.52
Slope = 0.26
Secreted protein level
mRNA level
C
PRE vs mean;
all SASP and non-SASP factors
Correl = 0.50
Slope = 0.27
SEN(REP) and SEN(XRA) vs mean;
all SASP and non-SASP factors
Correl = 0.30
Slope = 0.27
D
SASP factors;
all PRE, SEN(REP) and SEN(XRA) vs mean
Correl = 0.64
Slope = 0.37
Non-SASP factors;
all PRE, SEN(REP) and SEN(XRA) vs mean
Correl = 0.15
Slope = 0.05
