# Supplementary material for: Senescence-Associated Secretory Phenotypes Reveal Cell-Nonautonomous Functions of Oncogenic RAS and the p53 Tumor Suppressor
Source: PLoS Biol. 2008 Dec 2;6(12):e301. doi: 10.1371/journal.pbio.0060301 (PMC2592359; doi:10.1371/journal.pbio.0060301)

A

		SEN (REP;XRA)	SEN (RAS)	SEN (GSE > REP;XRA)	SEN (GSE > RAS)	REV
	p53 status	+	+	-	-	-
	RAS status	+	-	+	-	+
	Secretory profile extent	41	49	56	61	36
Profile comparison vs SEN (REP;XRA)	Overall conserved		32 (78 %)	39 (95 %)	35 (85 %)	21 (51 %)
	Overall conserved & further increased		17 (41 %)	24 (59 %)	24 (59 %)	9 (22 %)
	Uniquely increased		17	17	26	15

B

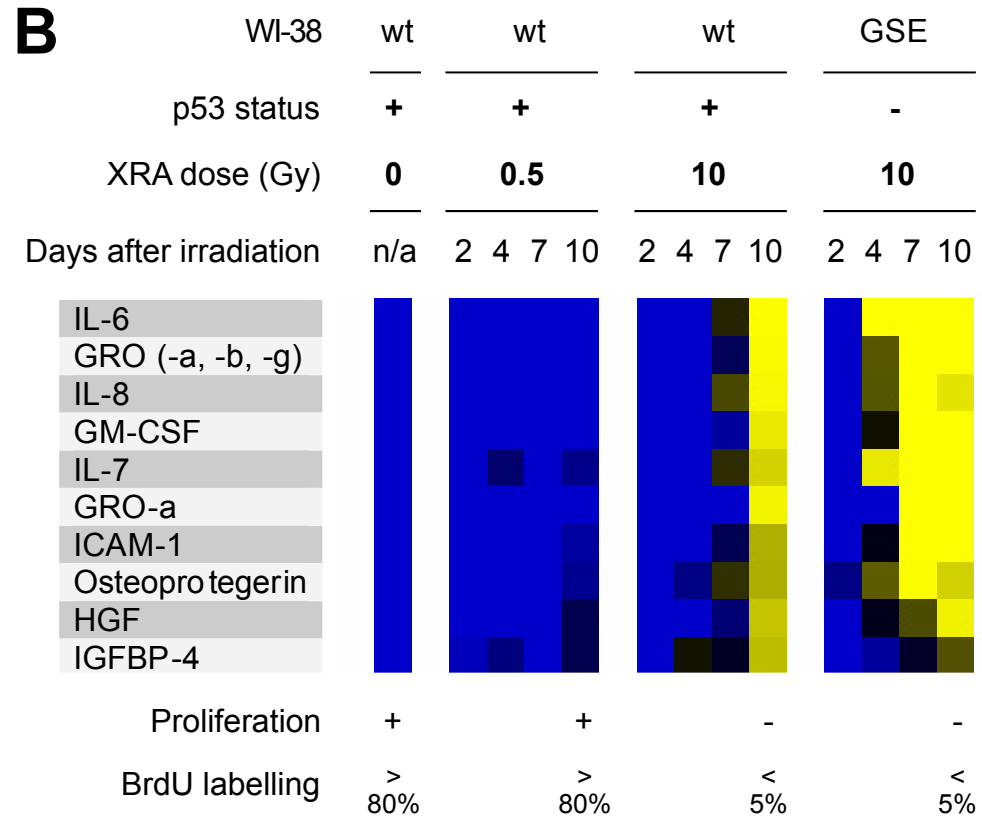

C

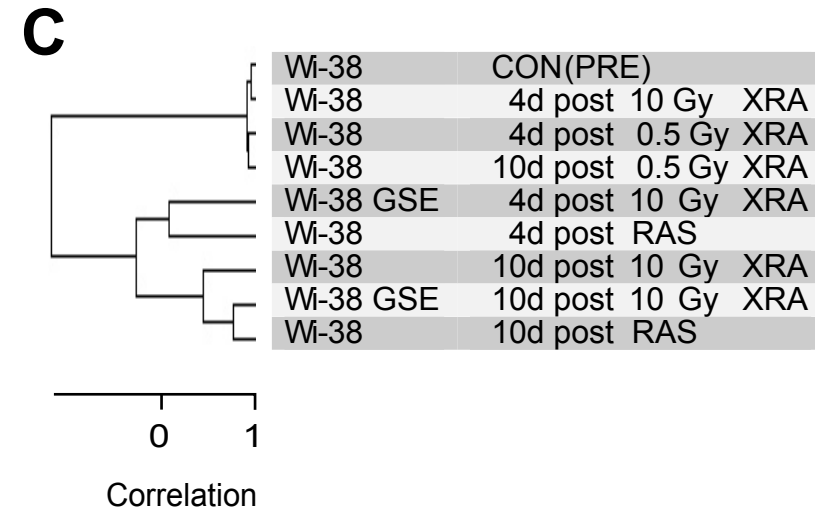

Supplement: Supplementary file 5 [file pbio.0060301.sg005.pdf]
