# Supplementary material for: Senescence-Associated Secretory Phenotypes Reveal Cell-Nonautonomous Functions of Oncogenic RAS and the p53 Tumor Suppressor
Source: PLoS Biol. 2008 Dec 2;6(12):e301. doi: 10.1371/journal.pbio.0060301 (PMC2592359; doi:10.1371/journal.pbio.0060301)
Supplement: Supplementary file 6 [file pbio.0060301.sg006.pdf]

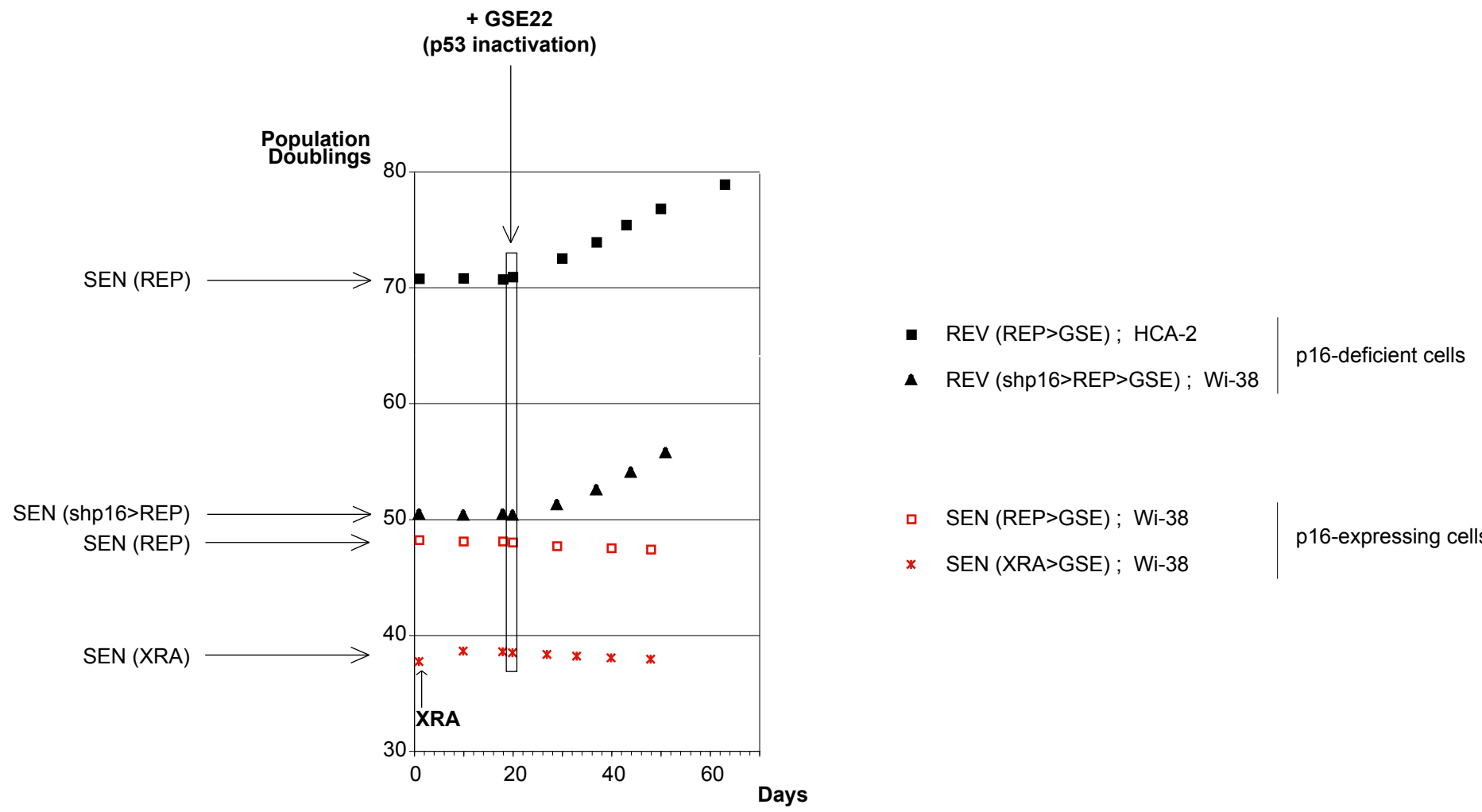
+ GSE22
(p53 inactivation)
Population Doublings
SEN (REP)
SEN (shp16>REP)
SEN (REP)
SEN (XRA)
XRA
80
70
60
50
40
30
0
20
40
60
Days
REV (REP>GSE) ; HCA-2
REV (shp16>REP>GSE) ; Wi-38
p16-deficient cells
SEN (REP>GSE) ; Wi-38
SEN (XRA>GSE) ; Wi-38
p16-expressing cells
