# Supplementary material for: Senescence-Associated Secretory Phenotypes Reveal Cell-Nonautonomous Functions of Oncogenic RAS and the p53 Tumor Suppressor
Source: PLoS Biol. 2008 Dec 2;6(12):e301. doi: 10.1371/journal.pbio.0060301 (PMC2592359; doi:10.1371/journal.pbio.0060301)
Supplement: Supplementary file 7 [file pbio.0060301.sd001.doc]

**Dataset S1 -- Key to Figure 1 and associated spreadsheets (Dataset S2,S3,S4)**

**• Figure 1 samples (supplemental spreadsheet: Dataset S2):**

Conditioned media samples from human fibroblast cultures, with descriptive information.

Sample: unique index name, representing a single replicate hybridized to a single array

Cell strain: self-explanatory

Growth state:

- PRE = presenescent and quiescent
- REP = senescent, due to replicative exhaustion
- XRA = senescent, 10 days after 10 Gy X-ray

Oxygen concentration: percent of atmospheric pressure at which cells were cultured

Category: the group into which each sample was pooled for averaging replicates (see next spreadsheet)

**• Figure 1 averaging (supplemental spreadsheet: Dataset S3):**

The first group of columns contains the protein intensity data for each sample. Proteins are listed along the vertical dimension; samples along the horizontal dimension. Intensity data for each array has been normalized to allow comparisons between separate experiments: The raw intensity data has been linearly re-scaled, so that the average of the BLANK/NEG spots on each array corresponds to a normalized intensity of 0, and the average of POS spots on each array corresponds to a normalized intensity of 100000.

The second group of columns contains averages of samples from the same category (e.g., all samples categorized as “BJ SEN 20”); the column headings correspond to the “Category” information in the previous spreadsheet.

As mentioned in the text of the manuscript and discussion of Figure 1, the secretory profiles of cell strains derived from the same tissue type were highly correlated. We therefore pooled and averaged these data to simplify the display. We tabulated the correlations between classes of samples for the reader’s inspection, and present a table of the correlations in the same worksheet, below the main body of the data.

**• Figure 1 fold change calculations (supplemental spreadsheet: Dataset S4):**

The first group of columns contains the category-averaged protein intensity data from the previous spreadsheet; each column refers to the average of all samples from a unique combination of cell strain, growth state and oxygen concentration. The second group of columns contains the average intensity observed over all categories for each cell strain; this average intensity is used as a “virtual baseline” for the purpose of calculating fold-changes. The third group of columns contains the log_2_ fold-change (l2fc) of each category average divided by the virtual baseline for the appropriate cell strain.

The final group of columns contains the results of statistical analyses performed on the l2fc data. Details are as follows:

SEN-PRE = (average l2fc for all SEN samples) – (average l2fc for all PRE samples)

- this number was used to sort the proteins for display in the figure; higher values correspond to a higher level of relative induction in SEN samples

TTEST(3%) = Two-tailed T-test comparing the distribution of all SEN 3% data to all PRE 3% data; in other words, at 3% oxygen, is the protein expressed at significantly different levels in SEN cells than in PRE cells?

Significant(3%) = If the TTEST(3%) is <= 0.05, the value is 1 (yes).

TTEST(20%) = As for TTEST(3%), but for cells grown at 20% oxygen

Significant(20%) = If TTEST(20%) is <=0.05, the value is 1 (yes)

TTEST(all) = As above, but comparing all SEN to all PRE data, regardless of the oxygen concentration

Significant(all) = If TTEST(all) is <=0.05, the value is 1 (yes)

CRITERION = if the protein passes (p<=0.05) any of the three significance tests in the preceding TTEST columns, the value is 1. Proteins with a value of 1 in this column were considered to be significantly regulated by senescence, and were included in the display in Figure 1A.

Correlations between classes of samples are presented in in the same worksheet, below the main body of the data.
