# Supplementary material for: Senescence-Associated Secretory Phenotypes Reveal Cell-Nonautonomous Functions of Oncogenic RAS and the p53 Tumor Suppressor
Source: PLoS Biol. 2008 Dec 2;6(12):e301. doi: 10.1371/journal.pbio.0060301 (PMC2592359; doi:10.1371/journal.pbio.0060301)
Supplement: Supplementary file 8 [file pbio.0060301.sd005.doc]

**Dataset S5 -- Key to Figure 2 and 3, and associated spreadsheets (Dataset S6,S7,S8)**

**• Figure 2 and 3 samples (supplemental spreadsheet: Dataset S6):**

Conditioned media samples from human epithelial cultures, with descriptive information.

Sample: unique index name, representing a single replicate hybridized to a single array

Cell strain or line: self-explanatory (see also *S25*)

Growth state:

- PRE = presenescent and quiescent
- XRA = senescent, 10 days after 10 Gy
- MIT = senescent, 10 days after Mitoxantrone treatment

Oxygen concentration: percent of atmospheric pressure at which cells were cultured

Category: the group into which each sample was pooled for averaging replicates (see next spreadsheet)

**• Figure 2 and 3 averaging (supplemental spreadsheet: Dataset S7):**

The first group of columns contains the protein intensity data for each sample. Proteins are listed along the vertical dimension; samples along the horizontal dimension. Intensity data for each array has been normalized to allow comparisons between separate experiments: The raw intensity data has been linearly re-scaled, so that the average of the BLANK/NEG spots on each array corresponds to a normalized intensity of 0, and the average of POS spots on each array corresponds to a normalized intensity of 100.

The second group of columns contains averages of samples from the same category (e.g., all samples categorized as “PrEC PRE”); the column headings correspond to the “Category” information in the previous spreadsheet.

**• Figure 2 and 3 fold change calculations (supplemental spreadsheet: Dataset S8):**

The first group of columns contains the category-averaged protein intensity data from the previous spreadsheet; each column refers to the average of all samples from a unique combination of cell strain, growth state and oxygen concentration.

The second group of columns contains the average intensity observed over PRE and XRA categories for each cell strain; this average intensity is used as a “virtual baseline” for the purpose of calculating fold-changes.

The third group of columns contains the log2 results of each raw value normalized to its corresponding average baseline (e.g. log2 of “PrEC PRE 1” divided by PrEC Ave calculated in S13). The significantly altered secreted factors are then selected using a two-tailed T-test comparing the distribution of all SEN(XRA) data to all PRE data. A p-value lower than 0.05 (outlined in yellow) is then used as the criterion to determine whether or not a protein is expressed at significantly different levels in SEN cells than in PRE cells. If the protein passes (p<=0.05), the column following the TTEST column is assigned a value of 1.

The fourth group of columns contains the log_2_ fold-change (l2fc) of each category average divided by the virtual baseline for the appropriate cell strain.

The fifth group of columns contains the results of statistical analyses performed on the l2fc data. If a protein has been assigned a value of 1 by TTEST (third group of columns; final column), then the variation around the mean (fourth group of columns) is selected in yellow for being reported in the Fig 2A.

The final group of columns contains the results of the log_2_ fold-change (l2fc) of each category average divided by PRE baseline for the appropriate cell strain. This allows to compare the SEN(MIT) and the SEN(XRA), as presented in Fig 2B. The same TTEST selection criterion can be applied (values highlighted in yellow) to select for senescence-associated changes and compare senescence induced by irradation and mitoxatrone treatment (see Fig 2B).
