# Supplementary material for: Senescence-Associated Secretory Phenotypes Reveal Cell-Nonautonomous Functions of Oncogenic RAS and the p53 Tumor Suppressor
Source: PLoS Biol. 2008 Dec 2;6(12):e301. doi: 10.1371/journal.pbio.0060301 (PMC2592359; doi:10.1371/journal.pbio.0060301)
Supplement: Supplementary file 10 [file pbio.0060301.sd013.doc]

**Dataset S13 -- Key to Figure 6, and associated spreadsheets (Dataset S14,S15,S16)**

**• Figure 6 samples (supplemental spreadsheet: Dataset S14):**

Conditioned media samples from human fibroblast cultures, with descriptive information.

Sample: unique index name, representing a single replicate hybridized to a single array

Cell strain: self-explanatory

Growth state:

- PRE = presenescent and quiescent
- REP = senescent, due to replicative exhaustion
- XRA = senescent, 10 days after 10 Gy X-ray
- SEN = senescent as a result of some treatment other than replicative exhaustion or X-irradiation
- REV = formerly senescent cell “reverted” into proliferation (see text and Figure S6 for details)

Oxygen concentration: percent of atmospheric pressure

Comments: Any modification or treatment to which the sample was subjected

- “+lenti-{x}” indicates infection with a lentiviral vector expressing protein {x}
- “+lenti-sh-{y}” indicates infection with a lentiviral vector expression an shRNA against gene {y}
- “{z}->{w}” should be read as “treatment {z} was followed by treatment {w}”,

Category: the group into which each sample was pooled for averaging over replicates (see next spreadsheet). Note that fibroblast strains of similar tissue origin have been pooled in these categories:

- HEF = “human embryonic fibroblast”; IMR-90 and Wi-38
- HSF = “human skin fibroblast”; BJ and HCA-2

**• Figure 6 averaging (supplemental spreadsheet: Dataset S15):**

The first group of columns contains the protein intensity data for each sample. Proteins are listed along the vertical dimension; samples along the horizontal dimension. Intensity data for each array has been normalized to allow comparisons between separate experiments: The raw intensity data has been linearly re-scaled, so that the average of the BLANK/NEG spots on each array corresponds to a normalized intensity of 0, and the average of POS spots on each array corresponds to a normalized intensity of 100000.

The second group of columns contains averages of samples from the same category (e.g., all samples categorized as “HEF SEN 3”); the column headings correspond to the “Category” information in the previous spreadsheet.

**• Figure 6 fold change calculations (supplemental spreadsheet: Dataset S16):**

The first group of columns contains the category-averaged protein intensity data from the previous spreadsheet; each column refers to the average of all samples from a unique combination of cell type, growth state, oxygen concentration and treatment condition (e.g., viral infection). The second group of columns contains the average intensity observed for PRE 3% samples from a given cell type. This average intensity is used as a “virtual baseline” for the purpose of calculating fold-changes, i.e., fold changes are calculated using the PRE 3% value as the denominator. The third group of columns contains the log_2_ fold-change (l2fc) of each category average divided by the virtual baseline for the appropriate cell type.

The final group of columns is a repetition of the identically named columns from the spreadsheet “Figure 1A fold change calculations.” Proteins were chosen for inclusion in Figure 1A on the basis of the significance of the difference between the distributions of all SEN and all PRE samples (“CRITERION”) and then ordered on the basis of their relative induction in SEN vs. PRE samples. The same proteins are included in this figure; the statistics by which significance was evaluated are included here for reference.
