# Supplementary material for: Senescence-Associated Secretory Phenotypes Reveal Cell-Nonautonomous Functions of Oncogenic RAS and the p53 Tumor Suppressor
Source: PLoS Biol. 2008 Dec 2;6(12):e301. doi: 10.1371/journal.pbio.0060301 (PMC2592359; doi:10.1371/journal.pbio.0060301)
Supplement: Supplementary file 11 [file pbio.0060301.sd017.doc]

**Text S1. Supplemental Methods**

**Cells**. Human fibroblasts were obtained from the American Tissue Type Collection (BJ), Coriell Cell Repository (WI-38, IMR-90), O. Pereira-Smith (University of Texas Health Science Center, San Antonio; HCA2), and M. Stampfer (Lawrence Berkeley National Laboratory; HBF184). Epithelial cells were obtained from Lonza (PrEC normal prostate epithelial cells), M. Bissell (Lawrence Berkeley National Laboratory; SCp2, EpH4v), J. Gray and R. Neve (Lawrence Berkeley National Laboratory; human breast cancer cell lines) and the American Type Culture Collection (BPH1, RWPE, PC3). Embryo and breast fibroblasts were isolated from C57/Bl mice, as described (Parrinello et al., 2005; Parrinello et al., 2003). Human fibroblasts were cultured and made senescent as described (Dimri et al., 1995; Krtolica et al., 2001). For 3% oxygen studies, mouse cells were prepared and cultured as described (Parrinello et al., 2003); human cells were cultured in 3% oxygen for at least 4 doublings prior to use. To induce senescence by ionizing radiation, cells were cultured to confluence, X-irradiated (10 Gy), allowed to recover overnight, then trypsinized and split 1:3; cells were analyzed 10 d later, when they displayed the enlarged senescent morphology and expressed SA-Bgal (Dimri et al., 1995). In general, human cultures were considered presenescent if, when assayed at subconfluence in 10% serum, >70% of cells incorporated BrdU or ^3^H-thymidine over a 1 d interval and <15% expressed SA-Bgal; cultures were considered senescent if, under the same conditions, <10% of cells incorporated BrdU or ^3^H-thymidine and >75% were SA-Bgal positive. Mouse fibroblasts that senesced in 20% oxygen frequently displayed high BrdU or ^3^H-thymidine incorporation (~40%) and relatively low SA-Bgal staining (~65%), despite little or no growth (Parrinello et al., 2003). Mouse fibroblasts that senesced in 3% oxygen, by contrast, displayed BrdU/^3^H-thymidine labeling and SA-Bgal staining similar to senescent human cells. Presenescent cultures were made quiescent by culturing to confluence, which reduced the 1 d BrdU labeling index to <10%. Proliferative capacities (1 d BrdU or ^3^H-thymidine labeling indices) and %SA-Bgal values for cultures used in these studies are given in Table S1.

**Indirect co-culture assays**. Epithelial cells (3x10^4^ per 35 mm dish) were seeded onto tissue culture plates in growth medium supplemented with 1.375 mM CaCl_2_ at least 2 d prior to incubation with CM from PRE or SEN fibroblasts, normalized for equal cell number per ml. After 7 d, epithelial cell growth was measured by quantifying GFP or DAPI-stained nuclei, as previously described (Krtolica et al., 2001).

**ELISA and immunofluorescence**. ELISA reagents were from RD Systems to detect FGF-7/KGF (DKG00), GROα (DGR00), IL-6 (D6050), IL-8 (D8000C), and IL-12 (D1200), and RayBiotech to detect OPG (ELH-OPG-001). Antibodies used for immunostaining were from RD Systems to detect IL-6 (AF-206-NA), IL-7 (MAB207), and IL-8 (MAB208), from NeoMarkers to detect p16 (Ab6-JC8) and cytokeratins 8 and 18 (MS743S0), from CellSignaling to detect p53 (#9282) and E-cadherin (cat #4065), from BD Biosciences to detect β-catenin (cat #610153), from Santa Cruz Biotechnology to detect claudin-1 (sc17658), and Sigma to detect vimentin (V5255). After incubation with the appropriate fluorochrome-conjugated secondary antibody, coverslips were counterstained with DAPI to visualize nuclei.

**Invasion assays.** Assays were performed in modified Boyden chambers using 8-micron pore filter inserts for 24-well plates (BD Bioscience). Filters were coated with 12 µl ice-cold 20% basement membrane extract (Matrigel, BD Bioscience). Epithelial cells (5 x10^4^ or 7.5x10^4^) were added to the upper chamber in 200 µl serum-free medium, while the lower chamber was filled with 300 µl fibroblast CM. After incubation for 16-24 h, epithelial cells on the under side of the filter were fixed with 2.5% glutaraldehyde in phosphate-buffered saline and stained with 0.5% toluidine blue in 2% Na_2_CO_3_. Cells that remained in the gel or attached to the upper side of the filter were removed with cotton tips. Cells on the under side of the filter were counted using light microscopy.

**Recombinant proteins and blocking antibodies.** Recombinant proteins and blocking antibodies were obtained from RD Systems (unless otherwise noted): human GROα (275-GR; MAB275 and general blocking antibody for GROα/β/γ MAB276), IL-6 (206-IL; MAB2061), IL-8 (1645 from Sigma; MAB208), and mouse KC (453-KC; MAB4531 and AF-453-NA). A non-specific control antibody from RD Systems was used (Goat IgG AB-108-C).

**Human study and tissues**. Frozen sections (7 µm) were cut from OCT blocks, stained with Mayer’s hematoxylin, dehydrated in 100% ethanol and xylene, and used for laser-capture microdissection (LCM) using an Arcturus PixCell IIe microscope (Arcturus, Mountain View, CA).

**Quantitative real time polymerase chain reaction (RT-PCR) analyses of human prostate tumor tissue and cultured cells**. For RT-PCR, total RNA was isolated from laser-captured epithelium using the Picopure RNA isolation kit and from cultured cells using the RNAwiz kit. cDNA was synthesized using standard methods. Quantitative reverse transcription reactions were done in duplicate or triplicate using SYBR Green PCR master mix (Applied Biosystems, Foster City, CA) and analyzed using an Applied Biosystems 7700 sequence detector. Samples were normalized to the cycle threshold value obtained during exponential amplification of GAPDH, GUS or H1A. Control reactions with RNA or water as template did not produce significant amplification products. Primer sets were obtained from Applied Biosystems. Specific primers were: IL-6 (Hs 00174131 m1), IL-8 (Hs 00174103 m1), IL-1β (Hs 00174097 m1), GROα (Hs 00236937 m1), GROβ (Hs 00236966 m1), GROγ (Hs 00171061 m1), GM-CSF (Hs 00929873 m1), ICAM-1 (Hs 00164932 m1), Osteoprotegerin (Hs 00171068 m1), MCP-1 (Hs 00234140 m1), MCP-2 (Hs 99999026 m1), MCP-3 (Hs 00171147 m1), MCP-4 (Hs 99999076 m1), IGFBP-2 (Hs 00167151 m1), IGFBP-3 (Hs 00181211 m1), p16 (Hs 00233365 m1), p21 (Hs 00355782 m1), Cyclin A (Hs 00153138 m1), MCM3 (Hs 00172459 m1), PCNA (Hs 00686962 m1), IL-2 (Hs 00174114 m1).

**References.**

Dimri, G.P., Lee, X., Basile, G., Acosta, M., Scott, G., Roskelley, C., Medrano, E.E., Linskens, M., Rubelj, I., Pereira-Smith, O.M.*, et al.* (1995). A novel biomarker identifies senescent human cells in culture and in aging skin in vivo. Proc Natl Acad Sci USA *92*, 9363-9367.

Krtolica, A., Parrinello, S., Lockett, S., Desprez, P., and Campisi, J. (2001). Senescent fibroblasts promote epithelial cell growth and tumorigenesis: A link between cancer and aging. Proc Natl Acad Sci USA *98*, 12072-12077.

Parrinello, S., Coppe, J.P., Krtolica, A., and Campisi, J. (2005). Stromal-epithelial interactions in aging and cancer: senescent fibroblasts alter epithelial cell differentiation. J Cell Sci *118*, 485-496.

Parrinello, S., Samper, E., Goldstein, J., Krtolica, A., Melov, S., and Campisi, J. (2003). Oxygen sensitivity severely limits the replicative life span of murine cells. Nature Cell Biol *5*, 741-747.
