# Supplementary material for: Senescence-Associated Secretory Phenotypes Reveal Cell-Nonautonomous Functions of Oncogenic RAS and the p53 Tumor Suppressor
Source: PLoS Biol. 2008 Dec 2;6(12):e301. doi: 10.1371/journal.pbio.0060301 (PMC2592359; doi:10.1371/journal.pbio.0060301)
Supplement: Supplementary file 12 [file pbio.0060301.sd018.doc]

**Text S2 -- COMPUTATIONAL PROCESSING, ANALYSIS AND VALIDATION OF ANTIBODY ARRAYS**

**A. Human proteins:** A complete catalog of Entrez Gene IDs for all proteins corresponding to antibodies on the arrays is given in supplemental Table S2.

**B. Digitization and quantification of antibody arrays:** Each hybridized array was exposed on a Molecular Dynamics Storm 8600 phosphorimager for an interval suitable to generate an exposure on which no spots were saturated. Scanned images were read using ImageQuant 1.2 (Molecular Dynamics), defining spots with the grid tool and correcting for background by subtracting the local median (intensity of grid border pixels for each spot).

Volume reports for a given array were computationally processed as follows. For each protein interrogated by the array: (1) The signal intensity was calculated as the average of the pair of spots corresponding to the protein. Data from a protein for which the spots did not agree (for which the ratio of spot intensities was >1s from the median ratio for the array) were not considered in further analysis. (2) The average intensity of BLANK and NEG spots on the array was subtracted from the protein intensity. (3) The protein intensity was normalized for exposure time, batch antibody concentration and streptavidin specific activity by re-scaling against the average value of POS spots (which contain a constant amount of biotin) on the array.

This processing yields a numerical intensity for proteins that can be readily compared between arrays. As described below, the method is linear across a large dynamic range, based on dilutions of a sample hybridized to multiple arrays; accurate, based on validation against independent ELISA tests on the same samples; and reliable, based on comparisons of independent replication of samples as well as triplicate hybridizations of a single sample.

**C. Numerical and statistical methods:** Where samples were combined for comparison, the arithmetic average of normalized protein intensity was computed. We processed the data for analysis and visual display as follows. For each protein, we determined its median abundance measurement across all samples from the same cell type, and considered this value a “virtual baseline.” Each protein measurement in a given cell type was then normalized against this baseline, and expressed as a log_2_ fold change.

Comparisons between samples (or averaged groups of samples) are given as the log_2_ fold change (log_2_[protein intensity in sample A/protein intensity in sample B]) throughout the figures and text. Significant differences between measurements were determined by two-tailed T-test with an assumption of equal variance, and required *p* < 0.05.

**D. Linearity**. To determine the linear range of the antibody arrays using our detection protocol, a single sample containing known quantities of six recombinant proteins interrogated by the arrays was serially diluted (1:1, 1:2, and 1:5). The dilutions were incubated with separate arrays. Scan intensities from the arrays were correlated against the known relative protein levels in the input (here expressed as arbitrary units where the 1:1 dilution contains 100000 units of each protein):

| **Protein** | **1:1**  **(100000 U)** | **1:2**  **(50000 U)** | **1:5**  **(20000 U)** | **Correlation** |
| --- | --- | --- | --- | --- |
| FGF-7 | 16292.9 | 6980.68 | 6873.21 | 0.93 |
| GROa | 37341.6 | 25336.3 | 13798.9 | 0.99 |
| IL-12 p40 | 7046.59 | 6441.84 | 5778.73 | 0.99 |
| IL-6 | 12596.7 | 5960.21 | 5082.04 | 0.96 |
| IL-8 | 280924 | 151586 | 70504.9 | 1.00 |
| PDGF-BB | 1036930 | 257179 | 47417.9 | 0.98 |

From the dilution series, we conclude that the antibody array method used here yields linear results for raw intensity readings in the range between 5x10^3^ to 1x10^6^, a range into which the majority of readings fell.

We also serially diluted a sample of CM (1:2, 1:5, 1:10, 1:50) and incubated the dilutions to separate arrays. We then computed the correlation between the scan intensities for each protein and the known relative protein levels in the input, excluding proteins whose intensity fell below the average BLANK/NEG intensity on any array. The accompanying graph displays the relationship between this correlation and the lowest-observed scan intensity (from the 1:50 dilution) for a given protein.

The correlation is excellent for lowest-observed intensities >1000, though many of the less intense samples also correlate well. In our analysis, when comparing signal intensities between two samples, we required that at least one of the samples have an intensity >1000 in order to consider the comparison significant.

**E. Accuracy**. To determine the accuracy of our antibody array technique, we validated protein array readings against ELISA measurements taken on the same recombinant protein sample used for the Linearity test. Red digits indicate readings outside the linear range of the ELISA, based on standard curves performed for this experiment.

| **Protein** | **Scan 1:1** | **ELISA 1:1** | **Scan 1:2** | **ELISA 1:2** | **Scan 1:5** | **ELISA 1:5** | **Correlation** |
| --- | --- | --- | --- | --- | --- | --- | --- |
| FGF-7 | 16292.9 | 597 | 6980.68 | 283 | 6873.21 | 42 | 0.91 |
| GROa | 37341.6 | 129 | 25336.3 | 62 | 13798.9 | 39 | 0.97 |
| IL-12 p40 | 7046.59 | 22 | 6441.84 | 3 | 5778.73 | -3 | 0.95 |
| IL-6 | 12596.7 | 28 | 5960.21 | 13 | 5082.04 | 5 | 0.97 |
| IL-8 | 280924 | 235 | 151586 | 104 | 70504.9 | 45 | 1.00 |
| PDGF-BB | 1036930 | 1076 | 257179 | 447 | 47417.9 | 205 | 1.00 |

We conclude that the antibody array technique is comparably linear to ELISA, and comparably accurate.

**F. Reliability.** Three independent samples (404, 406, 408; WI-38, 3% oxygen, PRE) were hybridized to separate arrays. Sample 406 was split between three arrays (406a, 406b, 406c) and hybridized in triplicate. Additionally, the three samples were pooled and hybridized to two arrays (MixA, MixB). The replicates demonstrate that the antibody array technique introduces no greater error in measurement than the naturally occurring variation that exists between true biological replicates. The data from this series of hybridizations are given below.

| **PROTEIN** | **404** | **408** | **406a** | **406b** | **406c** | **MixA** | **MixB** |
| --- | --- | --- | --- | --- | --- | --- | --- |
| Acrp30 | 5970.73 | 7702.77 | 10422.00 | 6748.83 | 5942.69 | 6423.31 | 9468.43 |
| AgRP | 3305.80 | 2614.94 | 8248.53 | 2803.28 | 1275.90 | 3514.51 | 6640.05 |
| Amphiregulin | 812.14 | 2023.16 | 2643.90 | 2199.51 | 716.91 | 2078.62 | 2343.15 |
| Angiogenin | 1086.70 | 19521.10 | 7289.11 | 19664.10 | 19052.20 | 12851.70 | 2290.50 |
| Angiopoietin-2 | 1997.11 | 4567.19 | 3047.99 | 2817.95 | 2734.42 | 2693.85 | 3559.94 |
| Axt | 1094.41 | 2276.25 | 3470.93 | 2591.37 | 0.19 | 1517.55 | 3000.35 |
| BDNF | 9448.32 | 11596.90 | 12543.50 | 8535.22 | 11152.40 | 9269.62 | 7289.36 |
| bFGF | 1912.36 | 2893.66 | 3535.50 | 2252.82 | 3413.33 | 1859.81 | 1489.38 |
| BLC | 2677.59 | 13730.60 | 15366.50 | 13196.30 | 13855.10 | 12977.50 | 7511.61 |
| BMP-4 | 388.76 | 12112.30 | 9684.07 | 9370.24 | 14370.90 | 8215.44 | 3170.66 |
| BMP-6 | 964.11 | 3265.56 | 2822.60 | 1753.43 | 513.16 | 1411.20 | 2544.85 |
| b-NGF | 64.60 | 2056.43 | 1671.51 | 813.17 | 0.19 | 948.24 | 3807.72 |
| BTC | 984.19 | 2530.94 | 2034.66 | 2989.61 | 2821.91 | 1838.84 | 2727.78 |
| CCL-28 | 4268.77 | 7979.64 | 8979.89 | 5804.43 | 7865.73 | 4583.65 | 7269.58 |
| CK-beta-8-1 | 1830.06 | 6409.58 | 2954.43 | 3642.37 | 2955.53 | 4176.12 | 2751.64 |
| CNTF | 3956.97 | 2733.06 | 3100.84 | 2339.69 | 980.30 | 2361.54 | 3268.13 |
| CTACK | 514.64 | 6049.47 | 2936.13 | 5841.83 | 6569.56 | 4602.37 | 1178.63 |
| Dtk | 4301.14 | 3851.73 | 7408.26 | 2221.53 | 2924.20 | 3548.27 | 5267.92 |
| EGF | 967.11 | 3181.36 | 1616.50 | 2775.06 | 1753.75 | 2627.64 | 1351.93 |
| EGF-R | 294.84 | 3141.99 | 3333.75 | 4726.11 | 4424.42 | 2624.06 | 2449.80 |
| ENA-78 | 2154.65 | 5472.08 | 6321.07 | 4709.59 | 6566.80 | 5043.17 | 3521.25 |
| Eotaxin | 2052.87 | 3899.77 | 4396.61 | 5068.31 | 4933.83 | 3236.49 | 3343.42 |
| Eotaxin-2 | 1202.64 | 3539.79 | 1950.47 | 2871.89 | 868.55 | 2467.50 | 1747.43 |
| Eotaxin-3 | 7140.18 | 10574.90 | 7826.34 | 10316.40 | 12382.90 | 9566.87 | 7282.42 |
| Fas/TNFRSF6 | 1570.40 | 7011.79 | 2400.90 | 4659.04 | 2912.22 | 3204.39 | 2181.05 |
| FGF-4 | 2275.23 | 3802.28 | 2916.94 | 2280.66 | 1146.09 | 3012.34 | 3023.94 |
| FGF-7 | 1618.78 | 3792.72 | 4347.23 | 3572.90 | 2976.33 | 1744.85 | 2183.40 |
| FGF-9 | 2052.55 | 4003.13 | 5163.13 | 5280.44 | 5861.23 | 4088.69 | 2187.03 |
| Fit-3 ligand | 1224.64 | 4118.65 | 2482.06 | 4079.66 | 5331.96 | 3813.88 | 1785.89 |
| Fractalkine | 8655.61 | 4077.07 | 13386.20 | 4161.46 | 2899.89 | 3530.28 | 14272.80 |
| GCP-2 | 9216.20 | 22207.80 | 15091.50 | 18552.20 | 25587.70 | 19709.40 | 7101.78 |
| GCSF | 2900.41 | 4928.99 | 3273.58 | 4115.53 | 2768.53 | 2677.01 | 3688.99 |
| GDNF | 2409.75 | 2577.31 | 4536.67 | 2110.02 | 1610.82 | 1158.93 | 4312.42 |
| GITR | 425.91 | 3884.48 | 1865.34 | 2877.58 | 4413.04 | 2556.70 | 1775.48 |
| GITR-ligand | 1375.75 | 10471.10 | 3864.44 | 21641.80 | 14358.40 | 10833.60 | 8135.28 |
| GM-CSF | 906.00 | 2176.71 | 2261.18 | 4996.75 | 795.88 | 1633.80 | 2198.93 |
| GRO | 521.45 | 2402.00 | 1468.55 | 1766.68 | 643.14 | 427.61 | 1878.05 |
| GRO-alpha | 2874.39 | 32224.80 | 24677.30 | 72331.50 | 78789.50 | 27994.80 | 10382.80 |
| HCC-4 | 1027.28 | 1328.57 | 1323.22 | 402.39 | 2008.65 | 1152.13 | 468.15 |
| HGF | 1052.53 | 1691.46 | 1801.54 | 857.61 | 311.21 | 736.66 | 1204.50 |
| I-309 | 1049.36 | 2828.26 | 2650.45 | 2216.32 | 1696.61 | 2100.34 | 2236.08 |
| ICAM-1 | 162.72 | 494.29 | 1243.55 | 615.34 | 0.19 | 151.51 | 1100.35 |
| ICAM-3 | 3513.77 | 9017.82 | 9400.90 | 6506.29 | 9446.34 | 8805.83 | 5752.52 |
| IFN-gamma | 1396.44 | 4312.40 | 3408.52 | 6761.44 | 6853.05 | 7420.49 | 2435.13 |
| IGF-1 | 2086.33 | 3426.54 | 3037.47 | 2481.60 | 2517.09 | 3126.54 | 3202.28 |
| IGF-1 SR | 1799.95 | 6130.81 | 10541.20 | 6659.12 | 6668.63 | 5376.42 | 4431.91 |
| IGFBP-1 | 1679.03 | 6973.87 | 13424.00 | 6748.63 | 7163.70 | 4585.47 | 5126.76 |
| IGFBP-2 | 3154.13 | 10630.00 | 4578.02 | 9218.46 | 8508.90 | 10245.70 | 4245.39 |
| IGFBP-3 | 1072.97 | 4242.18 | 1819.17 | 4153.83 | 3791.57 | 1785.63 | 838.94 |
| IGFBP-4 | 2203.49 | 2489.83 | 4020.86 | 1910.70 | 1416.04 | 2585.51 | 2668.45 |
| IGFBP-6 | 958.88 | 1786.27 | 1833.96 | 1456.13 | 436.54 | 930.62 | 1825.67 |
| IL-1 R1 | 345.37 | 1690.81 | 2444.88 | 1308.45 | 0.19 | 1529.12 | 2419.60 |
| IL-1 R4/ST2 | 5438.50 | 12135.50 | 6868.78 | 9343.46 | 11947.20 | 11920.30 | 8322.70 |
| IL-10 | 1546.18 | 3246.74 | 3422.09 | 2344.96 | 3416.26 | 2138.98 | 1790.73 |
| IL-11 | 12779.70 | 19447.80 | 20237.20 | 17317.00 | 25396.10 | 14866.20 | 15305.30 |
| IL-12 p40 | 4740.04 | 11779.90 | 19117.30 | 10391.00 | 13956.00 | 12510.40 | 11257.20 |
| IL-12 p70 | 1163.25 | 1415.16 | 2749.13 | 702.68 | 9.22 | 600.88 | 2616.21 |
| IL-13 | 1871.17 | 3488.16 | 3216.85 | 3069.11 | 2436.26 | 2481.43 | 1833.72 |
| IL-15 | 320.71 | 2423.87 | 2091.71 | 1605.10 | 1176.95 | 1359.82 | 1422.00 |
| IL-16 | 980.61 | 2503.33 | 251.22 | 3018.77 | 1952.49 | 1191.16 | 508.93 |
| IL-17 | 1273.79 | 4306.96 | 3293.76 | 4312.53 | 6152.10 | 4655.00 | 1669.48 |
| IL-1-alpha | 2728.25 | 5125.01 | 5874.19 | 5816.09 | 6357.38 | 5059.83 | 3208.62 |
| IL-1-beta | 1160.36 | 1451.95 | 2263.07 | 890.95 | 79.38 | 773.44 | 2882.47 |
| IL-1-ra | 1842.26 | 1273.75 | 3815.96 | 2537.24 | 1646.96 | 905.62 | 1271.72 |
| IL-2 | 1183.05 | 1959.60 | 1791.12 | 1240.14 | 397.64 | 841.57 | 1925.61 |
| IL-2 R-alpha | 5327.78 | 5026.13 | 6790.98 | 6032.83 | 5655.16 | 4152.36 | 9005.17 |
| IL-3 | 1382.13 | 2803.60 | 2424.50 | 3707.62 | 2884.74 | 1490.77 | 1736.68 |
| IL-4 | 907.79 | 2897.75 | 1467.48 | 2279.50 | 1917.68 | 1736.38 | 1627.27 |
| IL-5 | 7848.12 | 9721.36 | 9190.44 | 18879.00 | 16832.00 | 11613.40 | 8670.48 |
| IL-6 | 1112.38 | 3397.27 | 2456.14 | 1652.62 | 1064.96 | 551.80 | 2621.16 |
| IL-6 R | 445.14 | 1768.36 | 245.79 | 1589.39 | 2621.81 | 894.10 | 763.58 |
| IL-7 | 2625.47 | 8045.78 | 4390.11 | 10584.10 | 6921.36 | 9229.60 | 4583.17 |
| IL-8 | 3001.74 | 6282.62 | 4450.66 | 5914.77 | 6977.58 | 6228.93 | 2548.28 |
| I-TAC | 6301.89 | 3922.19 | 5477.56 | 2793.41 | 4898.17 | 3656.66 | 8186.50 |
| Leptin | 694.94 | 3910.33 | 1953.02 | 2960.23 | 2207.02 | 3380.51 | 1136.19 |
| LIGHT | 8543.19 | 23318.10 | 20320.30 | 20965.40 | 24736.10 | 17611.30 | 11547.50 |
| Lymphotactin | 4200.97 | 37384.20 | 16562.60 | 37415.10 | 57801.50 | 37169.50 | 10548.10 |
| MCP-1 | 14841.20 | 21521.40 | 20268.50 | 21333.90 | 24967.20 | 22399.70 | 7784.93 |
| MCP-2 | 3766.13 | 18150.50 | 6167.17 | 15409.50 | 20046.40 | 14954.00 | 2535.08 |
| MCP-3 | 557.79 | 1506.00 | 2318.25 | 2113.12 | 2840.04 | 1851.80 | 1584.14 |
| MCP-4 | 4446.08 | 9423.34 | 4843.45 | 8211.89 | 7458.63 | 6677.76 | 4312.96 |
| M-CSF | 1103.63 | 2940.34 | 2016.51 | 2151.64 | 1251.13 | 3248.73 | 1485.16 |
| MDC | 2046.14 | 6155.43 | 3390.71 | 4073.67 | 6627.22 | 4867.12 | 2769.84 |
| MIF | 4916.34 | 6931.88 | 6317.72 | 4553.72 | 5538.62 | 4417.49 | 5728.17 |
| MIG | 3492.56 | 7705.31 | 5155.27 | 3695.51 | 6226.29 | 4090.67 | 3590.99 |
| MIP-1-alpha | 1855.11 | 5956.87 | 4292.57 | 6930.12 | 6327.44 | 5252.99 | 5255.13 |
| MIP-1-beta | 1457.50 | 2073.65 | 4316.62 | 3070.62 | 2195.89 | 1769.73 | 1732.14 |
| MIP-1-delta | 9336.54 | 7016.00 | 12409.90 | 2888.08 | 3202.86 | 3736.68 | 12490.20 |
| MIP-3-alpha | 3509.60 | 3972.20 | 5644.03 | 2981.87 | 2024.89 | 2808.67 | 2912.55 |
| MIP-3-beta | 9829.89 | 37391.20 | 23279.40 | 29398.90 | 36255.00 | 34171.10 | 12389.50 |
| MSP-alpha | 1325.15 | 6404.36 | 4883.98 | 5306.96 | 5978.14 | 3786.38 | 2461.12 |
| NAP-2 | 1416.28 | 2695.89 | 2897.62 | 2599.92 | 1432.82 | 2099.10 | 3426.47 |
| NT-3 | 1465.98 | 3641.20 | 2808.93 | 2647.14 | 1706.68 | 2759.12 | 2668.57 |
| NT-4 | 1323.91 | 10701.70 | 3610.42 | 8331.84 | 6554.85 | 8419.26 | 2810.41 |
| Oncostatin M | 1030.31 | 6914.14 | 4830.52 | 6475.00 | 7740.60 | 5960.99 | 2243.88 |
| Osteoprotegerin | 737.62 | 1858.10 | 4771.57 | 3284.42 | 6292.86 | 3627.26 | 2405.42 |
| PARC | 1054.87 | 1423.85 | 2845.09 | 673.71 | 147.86 | 610.81 | 2244.52 |
| PDGF-BB | 7872.60 | 7608.68 | 13681.70 | 6436.53 | 10054.60 | 7374.05 | 7525.30 |
| PIGF | 3097.02 | 7341.10 | 3609.74 | 7433.30 | 8637.77 | 5871.98 | 2088.73 |
| RANTES | 2436.88 | 3330.94 | 5352.55 | 4558.45 | 5857.24 | 4228.89 | 3819.85 |
| SCF | 2066.81 | 3241.90 | 2458.02 | 2104.12 | 4188.99 | 3192.92 | 1339.91 |
| SDF-1 | 928.83 | 1576.85 | 2305.65 | 798.92 | 156.69 | 451.49 | 1581.68 |
| sgp130 | 1544.52 | 2423.96 | 1479.73 | 3352.00 | 4315.07 | 4172.75 | 1436.92 |
| sTNF RI | 2827.74 | 7139.07 | 3306.30 | 7260.32 | 7636.56 | 5316.22 | 2050.80 |
| sTNF RII | 4866.69 | 40688.20 | 9512.62 | 38833.50 | 29870.00 | 34276.50 | 8699.80 |
| TARC | 36246.90 | 76323.80 | 23185.60 | 58011.40 | 80792.40 | 53379.50 | 16050.30 |
| TECK | 5913.32 | 10000.50 | 10192.40 | 9300.59 | 10542.80 | 7503.98 | 4858.68 |
| TGF-beta-1 | 5871.96 | 6426.09 | 11101.00 | 5429.73 | 9767.67 | 5595.44 | 8140.49 |
| TGF-beta-3 | 2963.95 | 6143.98 | 5701.16 | 5180.48 | 4000.42 | 4773.22 | 4015.80 |
| Thrombopoietin | 2822.46 | 4340.09 | 4273.48 | 4152.61 | 1101.83 | 3029.33 | 2902.34 |
| TIMP-1 | 2214.00 | 4041.69 | 4148.18 | 3228.72 | 2179.49 | 2769.32 | 2792.22 |
| TIMP-2 | 1306.03 | 4453.12 | 2327.80 | 3093.60 | 4068.60 | 2801.54 | 2104.86 |
| TNF-alpha | 538.25 | 1299.61 | 1484.76 | 1090.77 | 0.19 | 872.93 | 2085.40 |
| TNF-beta | 2941.96 | 4520.65 | 4679.04 | 2654.65 | 2127.11 | 2740.50 | 3456.17 |
| TRAIL-R3 | 6303.16 | 7230.94 | 10377.40 | 8054.38 | 5522.87 | 8173.07 | 8819.48 |
| TRAIL-R4 | 0.17 | 3162.45 | 2197.81 | 1838.96 | 2328.09 | 1291.44 | 992.53 |
| uPAR | 897.48 | 5387.36 | 1817.77 | 2634.52 | 455.13 | 635.55 | 1970.53 |
| VEGF | 630.40 | 2905.14 | 1153.75 | 1778.09 | 830.91 | 2752.27 | 1686.18 |
| VEGF-D | 1035.33 | 3723.65 | 2716.08 | 2836.43 | 1897.82 | 873.26 | 2406.25 |
