# Supplementary material for: Senescence-Associated Secretory Phenotypes Reveal Cell-Nonautonomous Functions of Oncogenic RAS and the p53 Tumor Suppressor
Source: PLoS Biol. 2008 Dec 2;6(12):e301. doi: 10.1371/journal.pbio.0060301 (PMC2592359; doi:10.1371/journal.pbio.0060301)
Supplement: Supplementary file 13 [file pbio.0060301.st001.doc]

**Table S1. Presenescent and senescent cells characteristics.**

Human cells were obtained, cultured, labeled with BrdU or ^3^H-thymidine for 24 h and stained for SA-Bgal, as described (Beausejour et al., 2003; Dimri et al., 1995; Itahana et al., 2002; Itahana et al., 2003). Where indicated, cells were made quiescent by culturing until just confluent (quiescent) or cultured until replicative exhaustion (REP; no detectable growth over a 1 mo interval). Alternatively, cells were X-irradiated (XRA; 10 Gy) or infected with the indicated lentiviruses, as described in Methods. LI = % BrdU or ^3^H-thymidine-positive cells. % SA-bGAL = % senescence-associated beta-galactosidase positive cells. NT = not tested. Each LI and SA-Bgal value is representative of 2-8 experiments, which were used for array analyses. * = growing cells were were not used for array analyses.

| **HUMAN Cell Strain** | **Description / Treatment** | **%O_2_** | **Designation** | **Growth status** | **p53 status** | **LI** | **% SA-bGAL** |
| --- | --- | --- | --- | --- | --- | --- | --- |
|  |  |  |  |  |  |  |  |
| WI-38 | Embryonic lung fibroblast* | 3 | PRE | Growing | WT | 89 | 3 |
|  | Embryonic lung fibroblast* | 20 | PRE | Growing | WT | 84 | 4 |
|  | growth to confluence | 3 | PRE | Quiescent | WT | 10 | NT |
|  | growth to confluence | 20 | PRE | Quiescent | WT | 8 | NT |
|  | + control lentivirus* | 3 | PRE | Growing | WT | 88 | 5 |
|  | + control lentivirus* | 20 | PRE | Growing | WT | 81 | 4 |
|  | + control lentivirus, growth to confluence | 3 | PRE | Quiescent | WT | 9 | NT |
|  | + control lentivirus, growth to confluence | 20 | PRE | Quiescent | WT | 7 | NT |
|  | + GSE lentivirus* | 3 | PRE (GSE) | Growing | deficient | 93 | 3 |
|  | + GSE lentivirus, growth to confluence | 3 | PRE (GSE) | Quiescent | deficient | 10 | NT |
|  | growth to replicative exhaustion | 3 | SEN (REP) | Senescent | WT | 4 | 92 |
|  | growth to replicative exhaustion | 20 | SEN (REP) | Senescent | WT | 3 | 93 |
|  | + X-irradiated | 3 | SEN (XRA) | Senescent | WT | 5 | 90 |
|  | + X-irradiated | 20 | SEN (XRA) | Senescent | WT | 2 | 94 |
|  | + mitoxantrone | 3 | SEN (MITO) | Senescent | WT | 5 | 91 |
|  | + RAS lentivirus | 3 | SEN (RAS) | Senescent | WT | 5 | 93 |
|  | + p16 lentivirus | 3 | SEN (p16) | Senescent | WT | 11 | 83 |
|  | + sh-p16 lentivirus, growth to SEN (REP) | 20 | SEN (shp16>REP) | Senescent | WT | 4 | 91 |
|  | + GSE lentivirus, growth to SEN (REP) | 3 | SEN (GSE>REP) | Senescent | deficient | 4 | 94 |
|  | + GSE lentivirus, X-irradiated | 3 | SEN (GSE>XRA) | Senescent | deficient | 3 | 95 |
|  | + GSE lentivirus, + RAS lentivirus | 3 | SEN (GSE>RAS) | Senescent | deficient | 5 | 93 |
|  | growth to REP, + GSE lentivirus | 3 | SEN (GSE>REP) | Senescent | deficient | 4 | 90 |
|  | + sh-p16 lentivirus, growth to REP, +GSE lentivirus* | 3 | REV (shp16>REP>GSE) | Growing | deficient | 77 | 9 |
|  | + sh-p16 lentivirus, growth to REP, +GSE lentivirus | 3 | REV (shp16>REP>GSE) | Quiescent | deficient | NT | NT |
|  |  |  |  |  |  |  |  |
| IMR-90 | Embryonic lung fibroblast* | 3 | PRE | Growing | WT | 91 | 3 |
|  | Embryonic lung fibroblast* | 20 | PRE | Growing | WT | 88 | 4 |
|  | growth to confluence | 3 | PRE | Quiescent | WT | 7 | NT |
|  | growth to confluence | 20 | PRE | Quiescent | WT | 9 | NT |
|  | growth to REP | 3 | SEN (REP) | Senescent | WT | 3 | 94 |
|  | + X-irradiated | 3 | SEN (XRA) | Senescent | WT | 3 | 93 |
|  | + RAS lentivirus | 3 | SEN (RAS) | Senescent | WT | 5 | NT |
|  | + GSE lentivirus, X-irradiated | 3 | SEN (GSE>XRA) | Senescent | deficient | 5 | 94 |
|  |  |  |  |  |  |  |  |
| HCA2 | Neonatal foreskin fibroblast* | 3 | PRE | Growing | WT | 89 | 4 |
|  | growth to confluence | 3 | PRE | Quiescent | WT | 8 | NT |
|  | growth to REP | 3 | SEN (REP) | Senescent | WT | 4 | 91 |
|  | + X-irradiation | 3 | SEN (XRA) | Senescent | WT | 6 | 89 |
|  | + mitoxantrone | 3 | SEN (MITO) | Senescent | WT | 3 | 91 |
|  | growth to REP, + GSE lentivirus* | 3 | REV (REP>GSE) | Growing | deficient | 86 | 9 |
|  | growth to REP, + GSE lentivirus | 3 | REV (REP>GSE) | Quiescent | deficient | NT | NT |
|  |  |  |  |  |  |  |  |
| BJ | Neonatal foreskin fibroblast* | 3 | PRE | Growing | WT | 85 | 4 |
|  | growth to REP | 3 | SEN (REP) | Senescent | WT | 3 | 87 |
|  | + X-irradiation | 3 | SEN (XRA) | Senescent | WT | 5 | 89 |
|  | growth to REP, + GSE lentivirus* | 20 | REV (REP>GSE) | Growing | deficient | 82 | NT |
|  | growth to REP, + GSE lentivirus | 20 | REV (REP>GSE) | Quiescent | deficient | NT | NT |
|  |  |  |  |  |  |  |  |
| HBF184 | Adult breast fibroblast* | 3 | PRE | Growing | WT | 73 | 14 |
|  | Adult breast fibroblast* | 20 | PRE | Growing | WT | 80 | 11 |
|  | growth to confluence | 3 | PRE | Quiescent | WT | 6 | NT |
|  | growth to confluence | 20 | PRE | Quiescent | WT | 5 | NT |
|  | growth to REP | 3 | SEN (REP) | Senescent | WT | 5 | 81 |
|  | growth to REP | 20 | SEN (REP) | Senescent | WT | 4 | 84 |
|  | + X-irradiation | 3 | SEN (XRA) | Senescent | WT | 3 | 75 |
|  | + X-irradiation | 20 | SEN (XRA) | Senescent | WT | 3 | 77 |
|  |  |  |  |  |  |  |  |
| PrEC | Normal prostate epithelial cell | 20 | PRE | Growing | WT | 76 | 7 |
|  | growth to confluence | 20 | PRE | Quiescent | WT | 10 | NT |
|  | + X-irradiation | 20 | PRE | Senescent | WT | 3 | 87 |
|  |  |  |  |  |  |  |  |
| BPH1 | Prostate epithelial cell line | 20 | PRE | Growing | deficient | 82 | 8 |
|  | growth to confluence | 20 | PRE | Quiescent | deficient | 7 | NT |
|  | + X-irradiation | 20 | SEN (XRA) | Senescent | deficient | 2 | 95 |
|  | + mitoxantrone | 20 | SEN (MITO) | Senescent | deficient | 3 | NT |
|  |  |  |  |  |  |  |  |
| RWPE1 | Prostate epithelial cell line | 20 | PRE | Growing | deficient | 83 | 5 |
|  | growth to confluence | 20 | PRE | Quiescent | deficient | 8 | NT |
|  | + X-irradiation | 20 | SEN (XRA) | Senescent | deficient | 5 | 92 |
|  | + mitoxantrone | 20 | SEN (MITO) | Senescent | deficient | 3 | NT |
|  |  |  |  |  |  |  |  |
| PC3 | Prostate epithelial cell line | 20 | PRE | Growing | deficient | 90 | 2 |
|  | growth to confluence | 20 | PRE | Quiescent | deficient | 10 | NT |
|  | + X-irradiation | 20 | SEN (XRA) | Senescent | deficient | 2 | 94 |
|  | + mitoxantrone | 20 | SEN (MITO) | Senescent | deficient | 2 | 91 |

Beausejour, C.M., Krtolica, A., Galimi, F., Narita, M., Lowe, S.W., Yaswen, P., and Campisi, J. (2003). Reversal of human cellular senescence: roles of the p53 and p16 pathways. EMBO J *22*, 4212-4222.

Dimri, G.P., Lee, X., Basile, G., Acosta, M., Scott, G., Roskelley, C., Medrano, E.E., Linskens, M., Rubelj, I., Pereira-Smith, O.M.*, et al.* (1995). A novel biomarker identifies senescent human cells in culture and in aging skin in vivo. Proc Natl Acad Sci USA *92*, 9363-9367.

Itahana, K., Dimri, G.P., Hara, E., Itahana, Y., Zou, Y., Desprez, P.Y., and Campisi, J. (2002). A role for p53 in maintaining and establishing the quiescence growth arrest in human cells. J Biol Chem *277*, 18206-18214.

Itahana, K., Zou, Y., Itahana, Y., Martinez, J.L., Beausejour, C., Jacobs, J.J., Van Lohuizen, M., Band, V., Campisi, J., and Dimri, G.P. (2003). Control of the replicative life span of human fibroblasts by p16 and the polycomb protein Bmi-1. Molec Cell Biol *23*, 389-401.
